# Porous Aromatic Framework Nanosheets Anchored with Lewis Pairs for Efficient and Recyclable Heterogeneous Catalysis

**DOI:** 10.1002/advs.202000067

**Published:** 2020-10-01

**Authors:** Qinghao Meng, Yihan Huang, Dan Deng, Yajie Yang, Haoyan Sha, Xiaoqin Zou, Roland Faller, Ye Yuan, Guangshan Zhu

**Affiliations:** ^1^ Key Laboratory of Polyoxometalate Science of Ministry of Education Northeast Normal University Renmin Avenue Changchun 130024 China; ^2^ Department of Materials Science and Engineering University of California Davis Davis CA 95616 USA; ^3^ Department of Chemical Engineering University of California Davis Davis CA 95616 USA

**Keywords:** heterogeneous catalysis, Lewis pairs, nanosheets, porous aromatic frameworks, porous organic frameworks

## Abstract

Lewis pairs (LPs) with outstanding performance for nonmetal‐mediated catalysis reactions have high fundamental interest and remarkable application prospects. However, their solubility characteristics lead to instability and deactivation upon recycling. Here, the layered porous aromatic framework (PAF‐6), featuring two kinds of Lewis base sites (N_Piperazine_ and N_Triazine_), is exfoliated into few‐layer nanosheets to form the LP entity with the Lewis acid. After comparison with various porous networks and verification by density functional theory (DFT) calculations, the N_Triazine_ atom in the specific spatial environment is determined to preferably coordinate with the electron‐deficient boron compound in a sterically hindered pattern. LP‐bare porous product displays high catalytic activity for the hydrogenation of both olefin and imine compounds, and demonstrates ≈100% activity after 10 successful cycles in hydrogenation reactions. Considering the natural advantage of porous organic frameworks to construct LP groups opens up novel prospects for preparing other nonmetallic heterogeneous catalysts for efficient and recyclable catalysis.

## Graphical abstract

A porous aromatic framework featuring two kinds of Lewis base sites (N_Piperazine_ and N_Triazine_) is exfoliated into few‐layer nanosheets to form a Lewis pair (LP) group with the Lewis acid in situ. This LP‐bare porous product with the significant catalysis performance opens up prospects for preparing other nonmetallic heterogeneous catalysts.

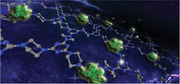

## 1 Introduction

Over the past decade, Lewis pairs (LPs) have prompted significant interest due to their broad applicability and outstanding efficiency in various nonmetal‐mediated catalysis conversions such as catalytic polymerization, asymmetric hydrogenation, reduction fields, and C—H bond activation.^[^ ^1^, ^2^, ^3^ ^]^ Unlike the conventional acid–base adduct, the crowded bulky blocks in LP integrations prevent Lewis pairs from forming a strong covalent bond and maintain their excitation reactivity. This augmented distance between acid and base centers provides access to cleave the central bond of small molecules (H_2_, CO_2_, etc.) to realize the metal‐free catalysis.^[^ ^4^, ^5^ ^]^ Although, some progresses with respect to selectivity, stability, and efficiency have been achieved for the practicability of LPs in hydrogenations.^[^ ^4^, ^5^ ^]^ However, most LP catalysts are soluble which will inevitably result in the dramatic loss in catalytic activity and functional stabilization upon recycling.^[^ ^6^, ^7^ ^]^ For current heterogeneous LP catalysts based on polymer matrices, the limited contact area restricts their catalytic efficiency to a large extent.^[^ ^8^, ^9^ ^]^ To address these issues, loading the LP catalysts into porous scaffolds would be an excellent route to achieve the desired catalytic activity and recyclability.^[^ ^10^ ^]^

Porous organic frameworks have been developed as innovative functional platforms for their robust skeletons constructed solely from light elements (H, B, C, N, and O) via covalent bonding.^[^ ^11^, ^12^ ^]^ Due to advanced reticular chemistry, one is able to prepare a diversity of porous frameworks with defined but tailored local structures for special functions.^[^ ^13^, ^14^, ^15^, ^16^, ^17^, ^18^ ^]^ Moreover, the large surface area, suitable pore size, and tunable structural constituents of porous organic frameworks provide excellent accessibility for the host–guest interaction between the architecture and substrate.^[^ ^19^, ^20^, ^21^, ^22^, ^23^, ^24^, ^25^, ^26^, ^27^ ^]^ By virtue of these innate strengths, a customized structure with B or N constituents may provide the sterically hindered Lewis acid/base groups for the postdecoration to realize the LP units. Nevertheless, there is only one case reported up to now that decorates the frustrated lewis pairs (FLP) groups in amorphous networks. Incorporation of FLP entity in an ordered structure to elaborate the formation mechanism of FLP units in porous skeleton is urgently demanded to guide the future works, which is still blank at present.

## 2 Results and Discussion

**Scheme 1:**
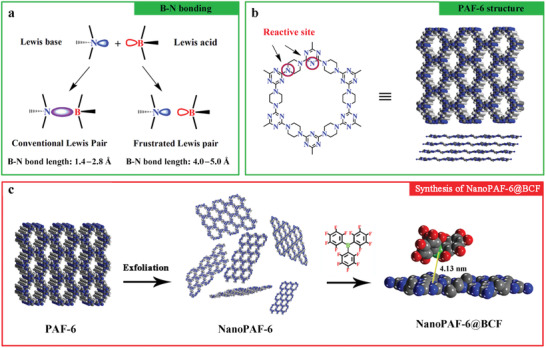
a) Classical and Lewis acid–base chemistry. b) PAF‐6 structure with two kinds of reactive sites. c) After exfoliation, PAF‐6 nanosheets react with BCF molecules into NanoPAF‐6@BCF.

In this work, the 2D porous aromatic framework (PAF‐6) with two kinds of Lewis base sites (N_Piperazine_ and N_Triazine_) is chosen as the research object.^[^ ^28^ ^]^ PAF‐6 was prepared in the A3 (cyanuric chloride) + B2 (piperazine) reaction system via a one‐step polymerization to build a monolith type hexagonal secondary framework. The structure of PAF‐6 is the *C*2/*m* space group and a unit cell with *a* = 20.68 Å, *b* = 17.80 Å, *c* = 4.68 Å, *β* = 79 Å. After exfoliation into few‐layer nanosheets, the expanded surface provides a considerable number of N atoms to interact with the strong Lewis acid (tris(pentafluorophenyl)borane, BCF) into the sterically encumbered Lewis pairs (**Scheme** **1**). In addition, some classical porous networks, including 2D CTF‐0, CTF‐1, TFM‐2, and LZU‐COF1 and 3D PAF‐2, are also selected as references to elucidate the structural factor for the formation of Lewis pairs. Adopting density functional theory (DFT) calculations using the Gaussian code,^[^ ^30^ ^]^ a mechanism is determined that the N_Triazine_ group with a specific spatial environment preferably binds with an acidic boron atom into the LP entity. Because of the full host–guest interaction of porous structure and excellent catalytic capability of the LP group, the LP‐bare porous material demonstrates good catalysis performance for the hydrogenation of both olefin and imine compounds and is easily recycled by washing with dichloromethane for several times without losing its catalytic activity.

PAF‐6 is synthesized according to our previous study and contains two kinds of N atoms (Scheme 1b) in the framework (N_Piperazine_ and N_Triazine_ denote the nitrogen atom in piperazine group and triazine group, respectively).^[^ ^28^ ^]^ Owing to the large dynamic diameter of the Lewis acid (BCF), PAF‐6 powder with 1.2 nm pore channel is treated by sonication in a cell homogenizer to prepare PAF‐6 nanosheets (NanoPAF‐6) which can be used to obtain a high aspect ratio to showcase the dimension‐related properties, including the contact area. Then, the synthetic procedure for PAF‐6 loading with BCF (NanoPAF‐6@BCF) was shown in Scheme 1c, the classical tris(pentafluorophenyl)borane (Lewis acid) is poured into the mixture of PAF‐6 nanosheets to react with the N atoms via acid–base interaction, named NanoPAF‐6@BCF (Scheme 1c).

**Figure 1:**
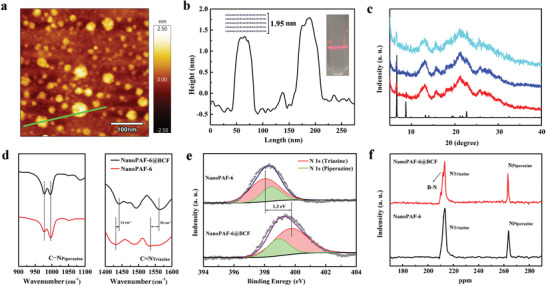
a) AFM image and b) height profile for NanoPAF‐6@BCF. c) PXRD for PAF materials. d) FTIR, e) N_1s_ XPS, and f) ^15^N NMR spectra for NanoPAF‐6 and NanoPAF‐6@BCF.

Scanning electron microscopy (SEM), transmission electron microscopy (TEM), and atomic force microscopy (AFM) are conducted to determine morphology changes of the PAF sample after exfoliation. Compared with the PAF‐6 precursor, NanoPAF‐6 and NanoPAF‐6@BCF exhibit a sheet‐like morphology with smaller size and reduced thickness (**Figure** **1** and Figures S1 and S2, Supporting Information). Furthermore, the distinct characteristics of nitrogen and boron elements in energy‐dispersive X‐ray spectroscopy (EDX) prove the uniform distribution of the Lewis acid/base groups in the PAF structures (Figures S3 and S4, Supporting Information). PAF‐6 materials were prepared through the copolymerization of piperazine and cyanuric chloride. The integrated flexible building unit (chair conformation of piperazine) into the backbone of PAF‐6 strongly weakens the affinity of interlayer stacking. Therefore, the 2D nanosheet can be easily exfoliated through the sonicating treatment.^[^ ^29^ ^]^ The thicknesses of NanoPAF‐6 and NanoPAF‐6@BCF are 1.8–2.0 nm (Figure 1a,b). Considering the distance between adjacent layers is ≈3.91 Å, this result suggests that NanoPAF‐6 and NanoPAF‐6@BCF consist of 4–5 atomic layers. These results, combined with the Tyndall effect for NanoPAF‐6@BCF (Figure 1b), demonstrate that the PAF‐6 particle has been successfully exfoliated into ultrathin 2D nanolayers. As shown in Figure 1c, the structures of PAF‐6, NanoPAF‐6, and NanoPAF‐6@BCF are verified through powder X‐ray diffraction (PXRD) analysis. All PAF samples exhibit a series of intense peaks at 6.64°, 8.70°, and 22.70°, revealing that NanoPAF‐6 and NanoPAF‐6@BCF preserve the periodic and extended hexagonal polygons after the PAF‐6 bulk is exfoliated into nanosheets and reacted with the Lewis acid (BCF).^[^ ^28^ ^]^

As illustrated in the Fourier transform infrared (FTIR) spectra, the absence of C–Cl stretching vibration in pristine PAF‐6 and NanoPAF‐6@BCF at 850 cm^−1^ confirms the completion of the coupling‐reaction (Figure S5, Supporting Information). The identical signal at 1103 cm^−1^, assigned to the C–N_piperazine_ bond before and after the addition of the BCF molecule, indicates that the N_piperazine_ fragment is not involved in the acid–base reaction. In contrast, several characteristic peaks centered at 1297 and 1487 cm^−1^ belong to the stretching vibration bonds of the piperazine and triazine core in NanoPAF‐6 (Figure 1d). There is a shift of the C=N_triazine_ vibration band from its original 1427 to 1441 cm^−1^ for NanoPAF‐6@BCF, this positive shift of the C=N vibration band indicates the interaction between B atom in BCF and N_Triazine_ atom, because nitrogen atom provides its electrons to boron atom leading to the decrease in the electron cloud density around the N atomic nucleus. The increase in the degree of less electron‐deficiency coupled with the increase in bond length results in the absorption peak of the C=N band move to a higher wavenumber which proving the interaction between the boron atom in the BCF molecule and the nitrogen atom in the triazine ring.

Similarly, X‐ray photoelectron spectroscopy (XPS) as an effective analysis method is performed to probe the structural compositions and chemical states of the N atom. Figure S6 (Supporting Information) portrays the B_1s_ XPS spectra at room temperature with the peaks ranging from 190.3 eV for BCF to 190.0 eV for NanoPAF‐6@BCF. As shown in Figure 1e, NanoPAF‐6 exhibits two kinds of N_1s_ XPS signals at 398.4 and 398.5 eV, corresponding to N_Piperazine_ and N_Triazine_, respectively. After interaction with the BCF molecules, the 1s N_Piperazine_ XPS remains unchanged and the bonding energy of N_Triazine_ changes from 398.5 to 399.8 eV, suggesting the Lewis pairs between the B center and the N_Triazine_ atom.^[^ ^31^ ^]^ The peaks located at 264 and 215 ppm are assigned to the N_Piperazine_ and N_Triazine_ atoms, respectively, in ^15^N NMR spectroscopy. The emerged N peak located at 212 ppm is derived from N_Triazine_ signal after the interaction between N_Triazine_ atom and BCF molecule (Figure 1f). All these results show that the N_Triazine_ atom reacts with the B atom (BCF molecule) in the NanoPAF‐6@BCF product.

N_2_ adsorption experiments conducted at 77 K (Figure S7a, Supporting Information) reveal that the exfoliated PAF‐6 nanosheets possess a larger Brunauer–Emmett–Teller (BET) surface area of 288 m^2^ g^−1^, exceeding the pristine value of the PAF‐6 sample (182.7 m^2^ g^−1^). This enlarged surface area was ascribed to the random displacement of 2D PAF‐6 layers increasing the gas contact space to some extent.^[^ ^32^, ^33^ ^]^ The NL‐DFT pore size distribution of NanoPAF‐6 centers at 1.2 and 4.2 nm (Figure S7b, Supporting Information). The emergence of 4.2 nm mesoporosity compared with PAF‐6 (1.2 nm) is attributed to gaps in the accumulated PAF nanosheets. As to NanoPAF‐6@BCF, there is a conspicuous decrease in the BET surface area (50 m^2^ g^−1^) and pore volume; meanwhile, the mesopore size of NanoPAF‐6@BCF reduces to 3.0 nm apparently, after the acid–base neutralization of BCF and PAF‐6 nanosheets. This phenomenon is caused by the guest molecules increasing the weight per structural unit and blocking the pore channels of NanoPAF‐6.

Thermogravimetric analysis (TGA) shows that PAF‐6 nanosheets possess two steps of weight loss. The first step is in the range of 300–350 °C due to the departure of the triazine core from the frameworks; the second one starts from 350 °C and ends at 500 °C on account of the decomposition of the piperazine linker (Figure S8, Supporting Information). Finally, there are almost no residues left after heating to 800 °C demonstrating the absence of any inorganic substances in the PAF‐6 architecture. As to NanoPAF‐6@BCF, it is stable up to 300 °C implying the excellent thermostability of the NanoPAF‐6@BCF product (Figure S9, Supporting Information). According to the elemental analysis by ICP‐MS, the boron amount is ≈4.64 mg g^−1^, corresponding to 0.43 mmol g^−1^ of BCF content in NanoPAF‐6@BCF. The boron element in NanoPAF‐6@BCF retains unchanged after immersion into nonpolar and polar solvents such as *n*‐hexane, dichloromethane (DCM), N,N‐dimethylformamide (DMF), dimethyl sulfoxide (DMSO), ethaonl (EtOH), and tetrahydrofuran (THF). In addition, there are no differences in the FTIR and XRD analysis between the original and final NanoPAF‐6@BCF, proving that NanoPAF‐6@BCF can maintain its structural integrity in various solvent environments (Figure S10, Supporting Information). The excellent thermostability and solvent stability ensure the wide applicability of NanoPAF‐6@BCF for practical utilization.

**Figure 2:**
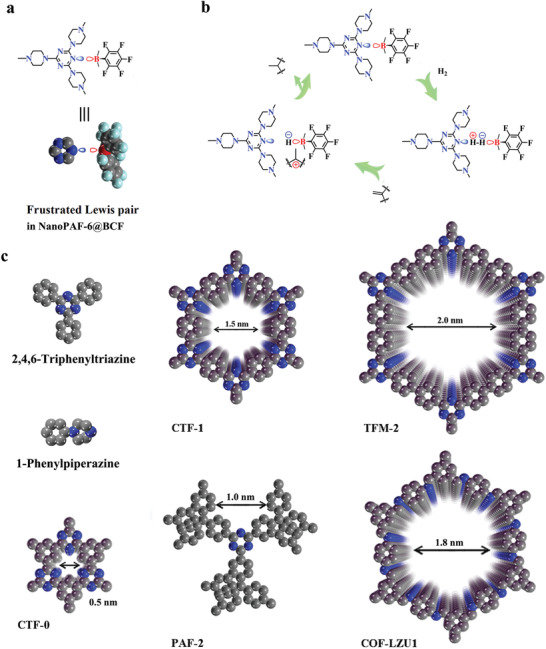
a) LP entity in NanoPAF‐6@BCF. b) Possible LP catalysis mechanism for cleaving the central bond of H_2_ molecule to realize the hydrogenation of olefin molecules. Reaction condition: each catalyst (≈0.01 mmol of catalytic sites) was put into 5 mL dichloromethane (DCM) at air atmosphere and stirred for 10 min. Then 1,1‐diphenylethene (0.2 mmol) was poured into the mixture, after purging with hydrogen flow for 5 min to remove the air the system was heated to 60 °C for 20 h under 10 bar H_2_ atmosphere. c) Various reference materials with N atoms in different environments.

Due to the equilibria governing of Lewis acid–base adducts, the LP group provides a high catalytic activity toward nonmetal‐mediated activation of “inert” small molecules, including H_2_ among others (**Figure** **2**). ^11^B NMR spectroscopy was carried out to investigate the reaction of LP group and activated H_2_ molecule, denoted as NanoPAF@BCF‐H_2_. As shown in Figure S11a (Supporting Information), there is a peak at −3.9 ppm for the parent NanoPAF@BCF in the ^11^B NMR spectrum. After the treatment in DCM under 10 bar H_2_ atmosphere at room temperature for 12 h, the ^11^B NMR spectrum shows a distinct variation with a peak centered at 4.9 ppm, confirming the formation of classical ammonium hydridoborate group.^[^ ^8^ ^]^ Furthermore, FTIR analysis was used to confirm the result of the formation of ammonium hydridoborate group in NanoPAF@BCF‐H_2_. As shown in Figure S11b (Supporting Information), the intensity for N–H stretching band (at 3007 cm^−1^) noticeably increased as compared with NanoPAF@BCF and NanoPAF@BCF‐H_2_. After adding ethylene‐based molecule (styrene) in NanoPAF@BCF‐H_2_ system, the (N—H) intensity restore to the original state, suggesting the existence of the LP‐H_2_ in the NanoPAF@BCF‐H_2_.^[^ ^8^ ^]^

The hydrogenation of 1,1‐diphenylethylene into 1,1‐diphenylethane under hydrogen environment, one representative LP‐catalyzed reaction, is selected as the model case to study the catalytic performance of LP‐based porous materials.^[^ ^34^, ^35^ ^]^ To eliminate any interference, we investigate the impact on pure BCF molecule, 1‐phenylpiperazine@BCF, 2,4,6‐Triphenyltriazine@BCF, PAF‐6, and PAF‐6 nanosheets loaded for the catalytic reaction (Figure 2c). The Lewis acid (BCF) produces a competitive edge for the hydrogenation of 1,1‐diphenylethylene and catalyses the dimerization of substrate. After it coordinated with N group, there is no dimerization of 1,1‐diphenylethene for all LP catalysts observed from the ^1^H NMR spectra. Meanwhile, 1‐phenylpiperazine@BCF, 2,4,6‐triphenyltriazine@BCF, PAF‐6, and PAF‐6 nanosheets possess no catalytic activity for the hydrogenation of 1,1‐diphenylethylene (entries 1–7 in Table S1 in the Supporting Information). Meanwhile, PAF‐6 powder is poured into the BCF solution, and ICP analysis reveals only 0.0064 mmol g^−1^ of BCF content in PAF‐6@BCF. EDX spectroscopy also agrees with the ICP conclusion that a small quantity of BCF molecules is incorporated on the PAF‐6 structure (Figure S3, Supporting Information). As calculated, PAF‐6@BCF without the exfoliation process provides the hydrogenated products with yield of 23% in 20 h (entry 8). It is exciting to note that NanoPAF‐6@BCF affords the hydrogenated products in excellent yields up to ≈91% (entry 9). This difference is due to the large dynamic diameter of BCF that restricts the amount entering into the PAF‐6 bulk channels. Thus, a handful of BCF molecules bind on the particle surface into LP groups, which seriously reduces the catalytic efficiency.

To study the formation mechanism, some control experiments are conducted using 2D networks (CTF‐0, CTF‐1, TFM‐2, and COF‐LZU1) and a 3D framework (PAF‐2) (Figure 2c).^[^ ^36^, ^37^, ^38^, ^39^ ^]^ With the same N_Triazine_ atom in the porous structure, the 2D networks CTF‐0, CTF‐1, and TFM‐2, featuring various pore sizes of 0.5, 1.5, and 2 nm, respectively, are prepared to serve as references according to previous reports (Figures S12–S17, Supporting Information). Additionally, CTF‐0, CTF‐1, and TFM‐2 are exfoliated into nanosheets to expand the contact area. After sonication and treatment with BCF, AFM imagery shows the thickness by length of exfoliated CTF‐0 (3.0 × 40 nm), CTF‐1 (1.6 × 50 nm), and TFM‐2 (1.2 × 70 nm). However, both porous organic material bulks and nanosheets of CTF‐0 (pore size of 0.5 nm) and CTF‐1 (pore size of 1.5 nm) with the same N_Triazine_ atom, especially CTF‐1 with a similar pore size as PAF‐6, reveal no catalytic capability (entries 10–15 in Table S1 of the Supporting Information). These results show that the N_Triazine_ atom plays a significant but not decisive role for the formation of the LP entity.

Maybe the twisted linker in the PAF‐6 network has a key impact by tuning the B—N bond length.^[^ ^40^ ^]^ Some other porous materials, TFM‐2 and 3D PAF‐2, are also examined to investigate the mechanism. TFM‐2 with large pore size of 2 nm would provide flexible living space to interact with BCF molecules and might lead to the increase of the B—N bond length. 3D PAF‐2 with a short range regular structure in ctn topology is prepared with microporosity of 1.02 nm; the small pore cavity benefits from the decrease in B—N bond length (Figure S18, Supporting Information). Simultaneously, 2D COF‐LZU1 bulk and COF‐LZU1 nanosheets (thickness by length: 2.5 × 50 nm) possess a sizeable analogous N_Triazine_ atom in a large pore channel (1.8 nm) to enrich the variations of the B—N interaction (Figure S19, Supporting Information). Using the above porous materials@BCF as catalysts, there was no product detected from the hydrogenation of 1,1‐diphenylethylene, implying failure of the sterically encumbered Lewis pairs (entries 16–23 in Table S1 in the Supporting Information). Based on the above results, we speculate that the PAF‐6 material can take the shape of the LP entity with the BCF molecule due to the N_Triazine_ atom in the specific twisted skeleton.

**Figure 3:**
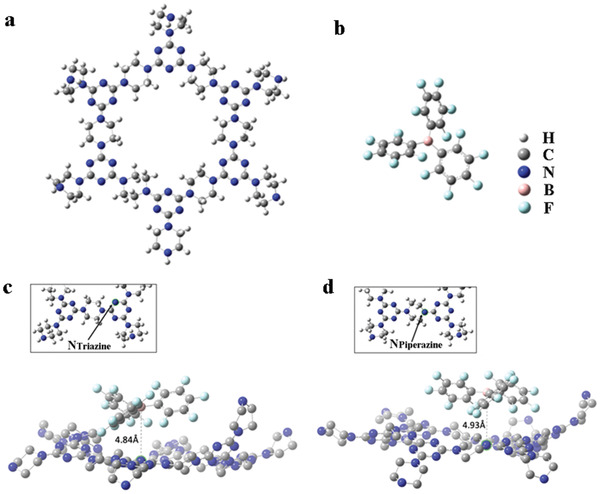
Relaxed structures: a) top view of PAF‐6 unit and b) BCF. UnitPAF‐6@BCF structure for two adsorption sites: c) B in BCF is 4.13 Å away from N_Triazine_ in PAF‐6; d) B in BCF is 4.55 Å away from N_Piperazine_ in PAF‐6. N_Piperazine_ and N_Triazine_ are highlighted in the figure, and hydrogens are removed only for visualization.

Normally, the basicity of N_Piperazine_ is stronger than that of N_Triazine_, however, in this context the Lewis acid (BCF) molecule coordinates to the N_Triazine_ atom in PAF‐6 network. This phenomenon could be due to the large volume of BCF molecule hampers the free coupling and preferentially binds to the N species from the out‐of‐plane orientation. DFT calculations are used to further determine the site of PAF‐6 on which BCF adsorbs (**Figure** **3**). All DFT calculations are performed by Gaussian code,^[^ ^30^ ^]^ with the hybrid functional B3LYP and the 6‐31G(d,p) basis set,^[^ ^41^, ^42^ ^]^ unless otherwise specified. Since the B‐N bond shows weak van der Waals forces, Grimme's D3 dispersion correction scheme^[^ ^43^ ^]^ was added in connection with B3LYP (B3LYP‐D3). The single layer of PAF‐6 is arranged in a hexagonal lattice with rings formed by alternating triazines and piperazines, so one ring with its six neighboring piperazines is chosen to be the unit structure and represents the 2D sheet in the DFT calculations. All dangling bonds on the edges are saturated with hydrogens to avoid unwanted edge reactions, and a total of 244 atoms are used in the system (the PAF‐6 unit, denoted as UnitPAF‐6, consists of 210 atoms, and the BCF unit consists of 34 atoms). This choice is a compromise for computational cost but should still represent the adsorption of NanoPAF‐6@BCF.

PAF‐6, BCF, and the complex structures are individually relaxed to their minimum energy states with all convergence criteria (maximum force 4.5 × 10^−4^ hartree bohr^−1^, RMS force 3 × 10^−4^ hartree bohr^−1^, maximum displacement 1.8 × 10^−3^ bohr, RMS displacement 1.2 × 10^−3^ bohr). Frequency calculations are then performed to evaluate the number of imaginary frequencies. Zero imaginary frequency guarantees that the structure is in a local energy minimum. Figure 3c,d shows the relaxed structures of UnitPAF‐6 and BCF. Two types of nitrogen (N_Piperazine_ and N_Triazine_) are present in UnitPAF‐6 for the adsorption. The adsorption energy comparison is calculated by Equation (1) and tabulated in **Table** **1**. For both cases, BCF is adsorbed out‐of‐plane. Energy comparison shows that N_Triazine_ is a more attractive site to the boron in BCF, so UnitPAF‐6@BCF complex is more likely to form through the B–N_Triazine_ interaction. In addition, in the complex products, the B–N_Triazine_ distance is 4.13 Å (Figure 3c), shorter than the B–N_Piperazine_ distance, which is 4.55 Å (Figure 3d), also indicating that the B–N_Triazine_ interaction is stronger than the B–N_Piperazine_ interaction. Adsorption energies on many other equivalent N_Piperazine_ and N_Triazine_ sites on UnitPAF‐6 are also calculated, and they follow the same trend as in Table 1, namely, that N_Triazine_ is more attracted to boron in BCF.

**Table 1:** Adsorption calculations of BCF on UnitPAF‐6

		Charge transfer [e^−^]	
Adsorption site^a)^	*E*_adsorption_ [kcal mol^−1^]	CHELPG	MK
N_Piperazine_	−23.748	0.080	0.050
N_Triazine_	−37.550	0.118	0.079

^a)^ Adsorption energies of BCF on UnitPAF‐6. Amount of charge transferred after BCF is adsorbed on the N_Piperazine_ or N_Triazine_ site on UnitPAF‐6. CHELPG and MK charge‐assigning schemes were used to calculate all the partial atomic charges.

In addition to adsorption energy comparison, the atomic charge distribution is also obtained using DFT. Since partial atomic charge is not an uniquely defined observable in quantum mechanics, and different charge schemes can yield significantly different partial atomic charge values,^[^ ^44^ ^]^ two charge‐assignment schemes—CHELPG^[^ ^45^ ^]^ and MK^[^ ^46^, ^47^ ^]^—are used to compare and verify the B–N charge transfer for the two possible sites. Both schemes assign partial charges to atoms in order to best fit the molecular electrostatic potential at a number of points around the molecule. The schemes are superior to the common Mullikan scheme^[^ ^48^, ^49^ ^]^ and are among the most robust partial charge schemes. After adsorption, the charge transferred from the two kinds of Lewis base sites (N_Piperazine_ and N_Triazine_ on UnitPAF‐6) to BCF are recorded in Table 1, for both CHELPG and MK schemes using B3LYP/6‐311G(d,p). The results show that upon adsorption on N_Piperazine_ site, there are 0.080 e^−^ and 0.050 e^−^ charge transferred between UnitPAF‐6 and BCF by CHELPG and MK respectively, and the amount of charge transferred between UnitPAF‐6 and BCF while adsorbing on the N_Triazine_ site is about 1.5 times more (0.118 e^−^ and 0.079 by e^−^ CHELPG and MK, respectively). Although the amount of charge transfer is small, the underlying trend still indicates that N_Triazine_ is more basic than N_Piperazine_ for the UnitPAF‐BCF interaction, and thus stronger bonding for the UnitPAF‐6@BCF complex on the N_Triazine_ site. The barrier of forming LP at N_Triazine_ site is 2.61 kcal mol^−1^ lower than at N_Piperazine_ site, using the Hartree–Fock (HF)^[^ ^50^ ^]^ method for computational efficiency. All these results show that the LP entity would be produced in the NanoPAF‐6@BCF structure because the specific N_Triazine_ atom in a twisted environment binds with BCF substance from the out‐of‐plane orientation.

To determine the local interaction between B and N, XPS and IR analyses were conducted. As shown in Figure S22 (Supporting Information), we synthesized the traditional Lewis acid–base adduct (Lutidine@BCF) as the reference.^[^ ^7^ ^]^ The reported distance between boron and nitrogen atoms is ≈1.6 Å (Figure S22a, Supporting Information), which is at the boundary of classical and frustrated Lewis pair. Correspondingly, as shown in Figure S22c (Supporting Information), the shift for B_1s_ binding energy is ≈1.25 eV (from 190.34 to 189.05 eV) in the XPS spectra because the acid–base adduct with a short boron and nitrogen distance increases the electron cloud density of B element leading to the drastically changed binding energy. As for NanoPAF‐6@BCF, the distance between boron and nitrogen atoms is calculated to be 4.13 Å (Figure S22b, Supporting Information), and the XPS shift is only 0.34 eV (Figure S22d in the Supporting Information, from 190.34 to 190.00 eV). This long distance between B and N atoms leads to the weak interaction between Lewis acid and Lewis base resulting in a small change of binding energy. According to previous reports, the 0.34 eV shift is within the scope of van der Waals forces.^[^ ^51^ ^]^ In the FTIR spectrum for traditional adduct (Lutidine@BCF), the C=N stretching vibration shifts by 27 cm^−1^ (Figure S22e, from 1447 to 1474 cm^−1^) which indicates the strong binding interaction between N and B elements. As for NanoPAF‐6@BCF, the shift for C=N stretching vibration is only 14 cm^−1^ (Figure S22f, Supporting Information, from 1427 cm^−1^ of NanoPAF‐6 to 1441 cm^−1^ of NanoPAF‐6@BCF). The variation for C=N band in NanoPAF‐6@BCF is much smaller than that in Lutidine@BCF which further proves the van der Waals forces between the B and N atoms in NanoPAF‐6@BCF.^[^ ^52^ ^]^

**Figure 4:**
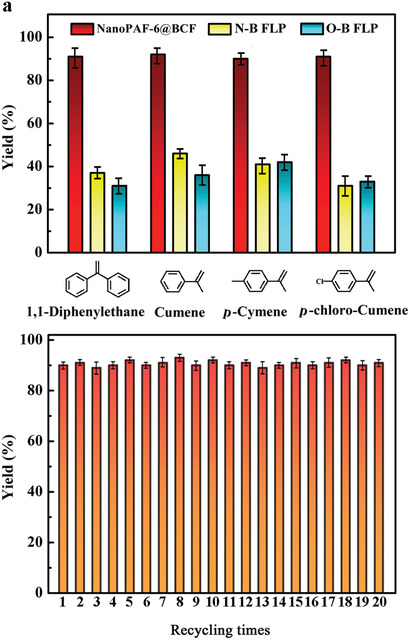
a) Catalysis performance of N–B LP, O–B LP, and NanoPAF‐6@BCF for various substrates, respectively. Reaction condition: each catalyst (NanoPAF‐6@BCF with ≈0.01 mmol of catalytic sites, 0.01 mmol N–B LP, and 0.01 mmol O–B LP) was put into 5 mL dichloromethane (DCM) at air atmosphere and stirred for 10 min. Then 1,1‐diphenylethene (0.2 mmol) was poured into the mixture, after purging with hydrogen flow for 5 min to remove the air the system was heated to 60 °C for 20 h under 10 bar H_2_ atmosphere. b) Recycling performance of NanoPAF‐6@BCF.

Encouraged by the excellent catalytic activity to 1,1‐diphenylethylene, some other olefin molecules, such as cumene, *p*‐cumene, and *p*‐chlorocumene, are utilized to test the catalytic activity of NanoPAF‐6@BCF. As observed in **Figure** **4**a, NanoPAF‐6@BCF affords the hydrogenated products in excellent (up to >91%) yields (Figure S20, Supporting Information). Emphatically, the catalysis capability of NanoPAF‐6@BCF clearly surpasses that of the homogeneous catalysts (N–B LP and O–B LP) by at least 140% under same conditions (Figure 4a).^[^ ^1^, ^2^, ^3^, ^4^ ^]^ This high activity is on account of two reasons: 1) the aromatic network enriches the phenyl molecule (1,1‐diphenylethylene) via *π*–*π* interaction and the concentrated substrates accelerate the metal‐free hydrogenation reaction (Table S2, Supporting Information);^[^ ^32^, ^33^ ^]^ 2) the accumulated PAF nanosheets provide the confined space surrounding their N_Triazine_ centers to restrict the rearrangement and escape of BCF molecules which enhances the chemical durability of LP groups in NanoPAF‐6@BCF.^[^ ^4^, ^5^, ^32^, ^33^ ^]^

The hydrogenation of unsaturated bonds has received wide interest because of the immense number of opportunities that exist to prepare high‐value products.^[^ ^53^ ^]^ Because NanoPAF‐6@BCF catalyst shows remarkable reactivity toward the activation of the H_2_ molecule, various imine compounds were chosen to investigate its catalysis capability for other hydrogenation reactions. The catalytic performances of NanoPAF‐6@BCF were evaluated by exposing each substrate molecule (0.2 mmol) to 1.2 equiv of HBPin and 20 mg catalyst (≈0.01 mmol of catalytic sites) in toluene for 24 h to complete the reaction. As shown in **Table** **2**, the reaction yields catalyzed by NanoPAF‐6@BCF are 92% for *N*‐*tert*‐butyl‐1‐phenylmethanimine, 93% for *N*‐benzylideneaniline, 90% for *N*‐benzylidene‐1‐phenylmethanamine, and 90% for *N*‐(1‐phenylethylidene)aniline (Table 2). These results clearly indicate that NanoPAF‐6@BCF has considerable capability for various nonmetal‐mediated catalysis conversions.

**Table 2:** Catalysis studies for reduction of C=N double bonds by NanoPAF‐6@BCF (reaction conditions: 20 mg NanoPAF‐6@BCF (≈0.01 mmol of catalytic sites) for heterogeneous catalytic reaction, 55 mg (0.43 mmol) HBPin, 0.2 mmol substrate, 3 mL toluene for 24 h)

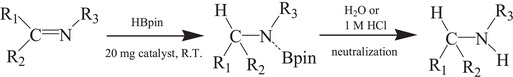		
Substrate	Entry	Yield [%]
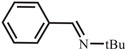	1	92
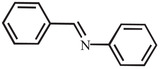	2	93
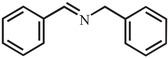	3	90
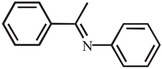	4	90

In addition to the outstanding catalytic activity, recyclability is an important factor. Hence, NanoPAF‐6@BCF as a heterogeneous catalyst is tested through the reduction of 1,1‐diphenylethylene. As shown in Figure 4b, while the exhausted NanoPAF‐6@BCF is recovered after centrifugation by CH_2_Cl_2_ for the next cycle; the conversion of 1,1‐diphenylethane exceeds 91% after twenty cycles. The XRD of NanoPAF‐6@BCF is consistent with fresh PAF‐6 (Figure S21, Supporting Information) and the content of BCF is maintained at ≈20.4%, which is close to the original content of 21.0%, indicating that the N atoms can efficiently prevent BCF molecules leaching to the reaction solution. The results demonstrate that NanoPAF‐6@BCF is an efficient heterogeneous catalyst that can undergo many cycles without obvious loss of catalytic activity, suggesting the wide generality and functional tolerance of the nonmetal mediated LP catalyst.

In summary, we design the Lewis pair decorated porous organic frameworks serving as heterogeneous catalysts which reveal significant hydrogenation catalytic properties. Through FTIR, XPS, and NMR analysis and comparison with other porous networks, the strong Lewis base N_triazine_ atom and the specific twisted structure of PAF‐6 are distinguished to provide the combined action for the formation of Lewis pairs. As determined by the DFT calculations, it is the pore size and chair structure of piperazine ring that impede the contact between B atom (BCF) and N atom (triazine ring) to achieve the specific LP entity in NanoPAF‐6@BCF. Based on the outstanding catalytic activity and notable recyclability of heterogeneous porous LP catalyst, our work lays a foundation for developing porous organic framework based LP catalysts for nonmetal‐mediated catalysis reactions.

## 3 Experimental Section

##### Preparation of PAF‐6 Nanosheets

In a typical experiment, dried PAF‐6 (5 mg) was put into 100 mL DCM. And the mixture was sonicated in the cell crusher for 50 min. After sedimentation for more than 15 h, the PAF‐6 nanolayers were collected by centrifuging the upper colloidal suspension, and dried under vacuum condition at 60 °C.

##### Synthesis of NanoPAF‐6@BCF

The NanoPAF‐6@BCF powder was prepared as follows: under N_2_ atmosphere, 100 mg BCF and 10 mg PAF‐6 were added into 10 mL dichloromethane and stirred for 10 h to make the BCF interacts with the PAF‐6 nanolayer. Then, the mixture was centrifuged and washed with THF for several times to remove the unloaded BCF. Finally, the product was dried under vacuum for 10 h.

##### Adsorption Energy Comparison

The potential energy of adsorbing BCF on UnitPAF‐6 is defined as the energy difference between the UnitPAF‐6@BCF complex product and the sum of isolated UnitPAF‐6 and BCF, Equation (1)

(1) $$\begin{array}{c} E_{\mathrm{adsorption}}=E_{\mathrm{UnitPAF}-6@\mathrm{BCF}}\;-E_{\mathrm{UnitPAF}-6}-E_{\mathrm{BCF}} \end{array}$$

##### Catalysis Procedure

A 20 mL Teflon‐lined Parr bomb equipped with a magnetic stir bar was charged with DCM (5 mL), NanoPAF‐6@BCF (20 mg with ≈0.01 mmol of active sites) and 1,1‐diphenylethene (0.2 mmol). The mixture was added into autoclave and filled with 10 bar H_2_. The system was heated to 60 °C for 20 h to complete the catalytic reaction and then centrifuged to separate the catalyst. All conversions were measured by ^1^H NMR integration and the product was purified by flash chromatography over silica gel to give the product.

##### Statistical Analysis

All the data displayed were representative of the results from multiple independent experiments. Differences among multiple groups were analyzed by one‐way ANOVA. All the data were presented as means ± standard deviation (SD), and comparison between groups were performed by unpaired *t*‐test. All experiments had a sample size of at least *n* = 10, and statistical analysis was performed using OriginPro 2016 (OriginLab Corporation).

## Conflict of Interest

The authors declare no conflict of interest.

## Supporting information

Supporting Information
