# Supplementary material for: Porous Aromatic Framework Nanosheets Anchored with Lewis Pairs for Efficient and Recyclable Heterogeneous Catalysis
Source: Adv Sci (Weinh). 2020 Oct 1;7(22):2000067. doi: 10.1002/advs.202000067 (PMC7675047; doi:10.1002/advs.202000067)
Supplement: Supplementary file 1 — Supporting Information [file ADVS-7-2000067-s001.pdf]

© 2020 Wiley-VCH GmbH

ADVANCED SCIENCE
Open Access

Supporting Information

for *Adv. Sci.*, DOI: 10.1002/advs.202000067

Porous Aromatic Framework Nanosheets Anchored with *Lewis* Pairs for Efficient and Recyclable Heterogeneous Catalysis

Qinghao Meng, Yihan Huang, Dan Deng, Yajie Yang, Haoyan Sha, Xiaoqin Zou, Roland Faller, Ye Yuan, and Guangshan Zhu**

Supporting Information

Porous Aromatic Framework Nanosheets Anchored with *Lewis* Pairs for Efficient and Recyclable Heterogeneous Catalysis

Qinghao Meng, Yihan Huang, Dan Deng, Yajie Yang, Haoyan Sha, Xiaoqin Zou, Roland Faller, Ye Yuan, and Guangshan Zhu**

Qinghao Meng, Dan Deng, Yajie Yang, Xiaoqin Zou, Ye Yuan, and Guangshan Zhu
Key Laboratory of Polyoxometalate Science of Ministry of Education, Northeast Normal University
Renmin Avenue, Changchun, 130024, China
E-mail: Yuany101@nenu.edu.cn; Zhugs@nenu.edu.cn
Yihan Huang
Department of Materials Science and Engineering, University of California, Davis, Davis, California 95616, United States.
Haoyan Sha and Roland Faller
Department of Chemical Engineering, University of California, Davis, Davis, California 95616, United States.

Keywords: *Lewis* pair; Nanosheets; Heterogeneous catalysis; Porous aromatic framework; Porous organic framework

Experimental Procedures

Materials: All starting materials were purchased from commercial suppliers and used without further purification unless otherwise noted. Piperazine and cyanuric chloride were purchased from Aldrich. And K_2CO_3, tris(pentafluorophenyl)borane (commonly abbreviated as BCF), and 1, 1-diphenylethene were purchased from Alfa Aesar.

Measurements: TG analysis was performed using a Netzch Sta 449c thermal analyzer system at the heating rate of 10 ℃min^{-1} in air atmosphere. FTIR spectra (film) were measured using a Nicolet Impact 410 Fourier transform infrared spectrometer. Solid-state ^{15}N-NMR spectrum was performed by a Bruker Avance 400 MHz Solid State NMR Spectrometer. The N_2 adsorption isotherms were measured on a Micromeritics ASAP 2010M analyzer. PXRD was performed by a Riguku D/MAX2550 diffractometer using Cu-Ka radiation, 40 kV, 200 mA with a scanning rate of 0.3 min^{-1} (2 theta). TEM was recorded using a JEOL JEM 3010 instrument with an acceleration voltage of 300 kV. SEM analysis was performed on a JEOS JSM 6700 system. AFM image and thickness were recorded using an OXFORD INSTURUMENTS CYPHER ES.

Synthesis of PAF-6*:* The synthesis method of PAF-6 was followed by previous report with a slightly improvement. 12 mmol piperazine, 12 mmol anhydrous K_2CO_3, and 50 mL anhydrous 1, 4-dioxane were charged into a 100 mL round-bottomed flask, and 8 mmol cyanuric chloride dissolved in 20 mL 1, 4-dioxane was added dropwise using a dropping funnel. The whole process above was conducted at 0 °C. Then the mixture was heated to 60 °C for 3 days. Finally, the product was collected by filtration and washed with methanol, water and tetrahydrofuran (THF) to remove the unreacted precursors entirely.[1]

Synthesis of CTF-0, CTF-1, TFM-2, LZU-COF1, PAF-2: These samples were synthesized according to the previous reports.[2-5]

Preparation of porous nanosheets: In a typical experiment, 5 mg dried porous materials (PAF-6, CTF-0, CTF-1, TFM-2, LZU-COF1) was put into 100 mL dichloromethane (DCM), respectively. And the mixture was sonicated in the cell crusher for 50 min. After sedimentation for more than 15 h, the Nano-layers were collected by centrifuging the upper colloidal suspension and dried under vacuum

condition at 60 °C. 1 mg porous nanosheets was dispersed in 10 mL ethyl alcohol and 0.1 mL above-mentioned solution was diluted for 10000 times.

Preparation of porous nanosheet@BCF: Under N_2 atmosphere, 100 mg BCF and 10 mg porous nanosheets (CTF-0, CTF-1, TFM-2, LZU-COF1) were added into 10 mL dichloromethane and stirred for 10 h to make the BCF interacts with the CTF-0 Nano-layer. Then, the mixture was centrifuged and washed with THF for several times to remove the unloaded BCF. Finally, the product was dried under vacuum for 10 h.

Preparation of PAF-2@BCF: The PAF-2@BCF powder was prepared as follow: under N_2 atmosphere, 100 mg BCF and 10 mg PAF-2 were added into 10 mL dichloromethane and stirred for 10 h to make the BCF interacts with the PAF-2. Then, the mixture was centrifuged and washed with THF for several times to remove the unloaded BCF. Finally, the product was dried under vacuum for 10 h.

Synthesis of 2,4,6-Triphenyltriazine: 2, 4, 6-Triphenyltriazine was prepared according to the previous article. Benzonitrile (0.26 mL, 2.48 mmol) with phosphorus pentoxide (P2O5, 1.5 g, 5.28 mmol) were charged in a glass ampoule in a glove box. After the same work-up procedures, 1, 3, 5-triphenyl-2, 4, 6-triazine (0.26 g, 86.6%) was afforded.

Synthesis of 2,4,6-Triphenyltriazine@BCF: The 2,4,6-Triphenyltriazine@BCF powder was prepared as follow: under N_2 atmosphere, 100 mg BCF and 10 mg 2,4,6-Triphenyltriazine were added into 10 mL dichloromethane and stirred for 10 h to make the BCF interacts with the 2,4,6-Triphenyltriazine. Then, the mixture was centrifuged and washed with THF for several times to remove the unloaded BCF. Finally, the product was dried under vacuum for 10 h.

Catalytic experiments:

For each catalyst: A 20 mL Teflon-lined Parr bomb equipped with a magnetic stir bar was charged with DCM (5 mL), each catalyst (~0.01 mmol of active sites) and 1, 1-diphenylethene (0.2 mmol). The mixture was added into autoclave and filled with 10 bar H_2. The system was heated to 60 °C for 20 h to complete the catalytic reaction and then centrifuged to separate the catalyst. The product was purified by flash chromatography over silica gel to give the product. All conversions were measured

by ^{1}H NMR integration and the product was purified by flash chromatography over silica gel to give the product.

Hydrogenated C=C double bonds: A 20 mL Teflon-lined Parr bomb equipped with a magnetic stir bar was charged with DCM (5 mL), nanoPAF-6@BCF (~0.01 mmol of active sites) and each substrate (0.2 mmol). The mixture was added into autoclave and filled with 10 bar H_2. The system was heated to 60 °C for 20 h to complete the catalytic reaction and then centrifuged to separate the catalyst. The product was purified by flash chromatography over silica gel to give the product. All conversions were measured by ^{1}H NMR integration and the product was purified by flash chromatography over silica gel to give the product.

1,1-Diphenylethane: ^{1}H NMR (600 MHz, $CDCl_3$) δ 7.35 – 7.11 (m, 10H), 4.15 (m, 1H), 1.63 (d, J=7.2, 3H);

p-Cymene: ^{1}H NMR (600 MHz, $CDCl_3$) δ 7.32 – 7.13 (m, 4H), 2.83 (m, 1H), 2.27 (s, 3H), 1.20 (d, J=7.6, 6H);

Isopropylbenzene: ^{1}H NMR (600 MHz, $CDCl_3$) δ 7.21–7.29 (m, 4H), 2.89 (m, 1H), 1.23 (d, J = 6.9 Hz, 6H);

1-Chloro-4-isopropylbenzene: ^{1}H NMR (600 MHz, $CDCl_3$) δ 7.05 – 7.23 (m, 5H), 2.87 (m, 1H), 1.25 (d, J=7.2, 3H).

Hydrogenated C=N double bonds: The reaction was carried out by adding nanoPAF-6@BCF (20 mg, ~0.1 mmol of active sites), HBPin (55 mg, 0.43 mmol), each substrate (1a, 56 mg, 0.35 mmol). Subsequently, the reaction was stirred for 24 h in the glove box and then centrifuged to separate the catalyst. The product was purified by flash chromatography over silica gel to give the product. All conversions were measured by ^{1}H NMR integration and the product was purified by flash chromatography over silica gel to give the product.

1-(4,4,5,5-Tetramethyl-1,3,2-dioxaboronia-2-yl)-N-benzyl-2-methylpropan-2-amine: ^{1}H NMR (600 MHz, $CDCl_3$) δ 1.13 (s, 9H), 1.18 (s, 12H), 3.67 (s, 2H), 7.17 (d, J = 7.3 Hz, 2H), 7.23 (t, J = 7.2 Hz, 1H), 7.27 (d, J = 7.4 Hz, 2H);

N-Benzylaniline: ^1H NMR (600 MHz, $CDCl_3$) δ 4.08 (s, 1H), 4.39 (s, 2H), 6.70 (d, J = 6.7 Hz, 2H), 6.79 (t, J = 6.8 Hz, 1H), 7.24 (t, J = 7.2 Hz, 2H), 7.34 (m, 1H), 7.43 (m, 4H);

Dibenzylamine: ^1H NMR (600 MHz, $CDCl_3$) δ 1.83 (s, 1H), 3.86 (s, 4H), 7.31 (m, 2H), 7.33-7.42 (m, 8H);

N-(1-Phenylethyl)aniline: ^1H NMR (600 MHz, $CDCl_3$) δ 7.65 and 7.61 (m, 4H); 7.51 (tt, 1H, J = 7.2 Hz, J = 1.3 Hz); 7.41 (dd, 1H, J = 8.6 Hz, J = 7.3 Hz); 6.98 (tt, 1H, J = 7.3 Hz, J = 1.1 Hz); 6.82 (dd, 1H, J = 8.6 Hz, J = 0.9 Hz); 4.77 (q, 3H, J = 6.7 Hz); 4.28 (br s, 1H); 1.76 (d, 3H, J = 6.7 Hz).

For traditional LPs: A 20 mL Teflon-lined Parr bomb equipped with a magnetic stir bar was charged with diethyl ether/*N*-methyldiphenylamine (0.01 mmol) and BCF (0.01 mmol) to synthesize the traditional O-B and N-B LPs, respectively, based on the previous report.[6,7] After removal the diethyl ether solution, DCM (5 mL) and 1, 1-diphenylethene (0.2 mmol) were poured into the bottle to react with the respective LP. The mixture was added into autoclave and filled with 10 bar H_2. The system was heated to 60 °C for 20 h to complete the catalytic reaction and then centrifuged to separate the catalyst. All conversions were measured by ^1H NMR integration and the product was purified by flash chromatography over silica gel to give the product.

Impact of substrate concentrations: We calculated the local concentration of substrate molecules in the pore channels and compared the catalytic activity of homogeneous catalyst at the same concentrations.

Sorption isotherm tests: The NL-DFT pore size distribution of NanoPAF-6 centers at 1.2 and 4.2 nm, and the dynamic diameter of 1,1-diphenylethylene is only 0.99 nm, PAF-6 provide enough space for the interaction with 1,1-diphenylethylene. Initially, the 1, 1-diphenylethene solution was prepared with the concentrations ranged from 0.001 to 0.06 mmol cm^{-3}; then 20 mg of NanoPAF-6@BCF powder was added to 5 mL each 1, 1-diphenylethene solution and stirred at ambient conditions for 2 hours. After filtration, the treated PAF sample was

removed and the supernatant was measured with UV spectroscopy to get the concentration of 1, 1-diphenylethene. The uptake in the PAF channels was determined by the equation:

$$Q_e = \frac{c_o - c_e}{m} \times V$$

Q_e (mmol g^{-1}) represents the 1, 1-diphenylethene uptake, C_0 and C_e are the initial and equilibrium concentrations, V is the volume of solution, and m is the weight of PAF powder.

The pore volume of NanoPAF-6@BCF was calculated to be 0.21 cm^3 g^{-1} based on the N_2 sorption calculation. As the initial concentration (C_0) of 1, 1-diphenylethene ranged from 0.001 to 0.06 mmol cm^{-3}, the determined concentration (Cv) in the PAF channels increased from 0.05 to 0.13 mmol cm^{-3}, correspondingly. The total content of catalytic sites in NanoPAF-6@BCF powder was 0.5 mmol g^{-1}, and the density of catalytic sites in PAF architecture was about 2.4 mmol cm^{-3}. Subsequently, homogeneous N-B LP catalyst with the same content of catalytic sites was used to conduct the conversion under the same conditions. As illustrated in the table below, NanoPAF-6@BCF dispersed in the 1, 1-diphenylethene solution with C_0 concentration revealed a similar catalytic performance as homogeneous N-B LP catalyst tested in the solution with C_v concentration. Therefore, we believe that the enhanced catalytic performance of the PAF catalyst is ascribed to the increased concentration in the PAF skeleton.

Impact of exfoliated thickness: We investigated the impact of the number of layers. PAF materials were treated by sonication in a cell homogenizer for respective 0, 10, 20, 50 min to prepare PAF-6 nanosheets (NanoPAF-6) with different thickness. As illustrated in the Table S3, the thickness of NanoPAF-6 sample ranged from bulk particle to 1.95 nm.

The size of BCF is larger than the pore size of PAF-6, based on the analysis of FTIR, XPS and theoretical calculation, the BCF is adhered to the surface of PAF-6 network. Through the ultrasound treatment, PAF-6 bulk was exfoliated into nanosheets to expose the active $N_{Triazine}$ core. The scaled-up

Lewis base interacted with BCF molecules leading to the increased number of exposed catalytic sites (Table S3). For the catalytic efficiency, it was decided by two parts in terms of the number of exposed catalytic sites and the local concentration of substrate. As illustrated in Table S3, we find that the yield increases linearly with the increase in the number of catalytic sites under the same condition. Therefore the underlying mechanism responsible for enhanced catalytic performances is that the exfoliated nanosheets increase the number of exposed catalytic sites.

Correspondingly, we found that the less the number of layers, the faster the catalytic process goes. There is no competitive edge for the dimerization of substrate observed. As calculated, PAF-6@BCF without the exfoliation process provides the hydrogenated products with yield of 23% in 20 h. And NanoPAF-6@BCF affords the hydrogenated products in excellent yields up to ~91%.

Recycle Experiment: A 20 mL Teflon-lined Parr bomb equipped with a magnetic stir bar was charged with DCM (5 mL), NanoPAF-6@BCF (20 mg with ~0.01 mmol of active sites) and 1, 1-diphenylethene (0.2 mmol). The mixture was added into autoclave and filled with 10 bar H_2. The system was heated to 60 °C for 20 h to complete the catalytic reaction and then centrifuged to separate the catalyst. NanoPAF-6@BCF was washed by DCM for 3 times and vacuumed for use in the next cycling catalysis experiment. The conversions measured by ^{1}H NMR integration and the product was purified by flash chromatography over silica gel to give the product.

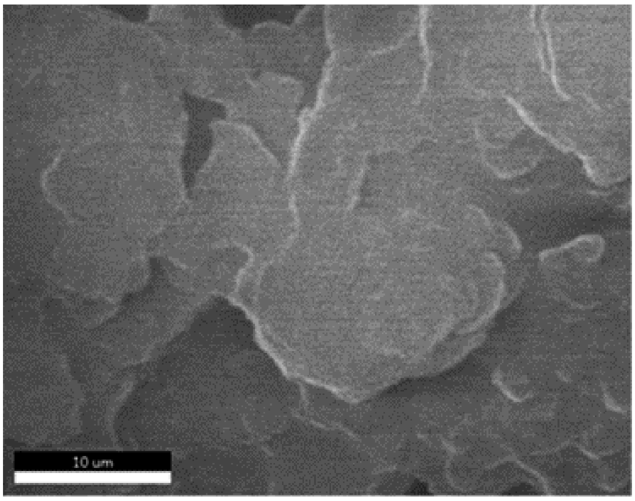

Figure S1. SEM image of NanoPAF-6@BCF.

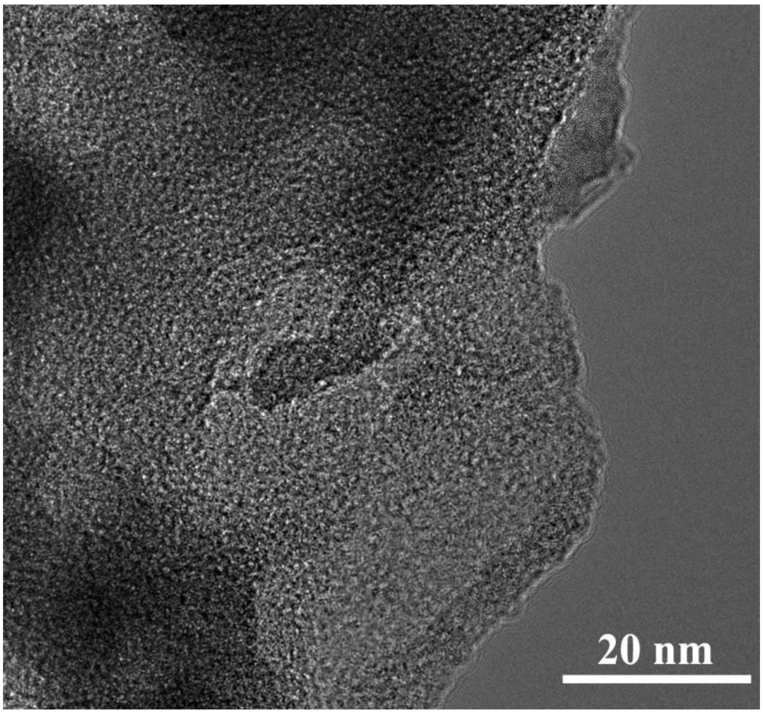

Figure S2. TEM image of NanoPAF-6@BCF.

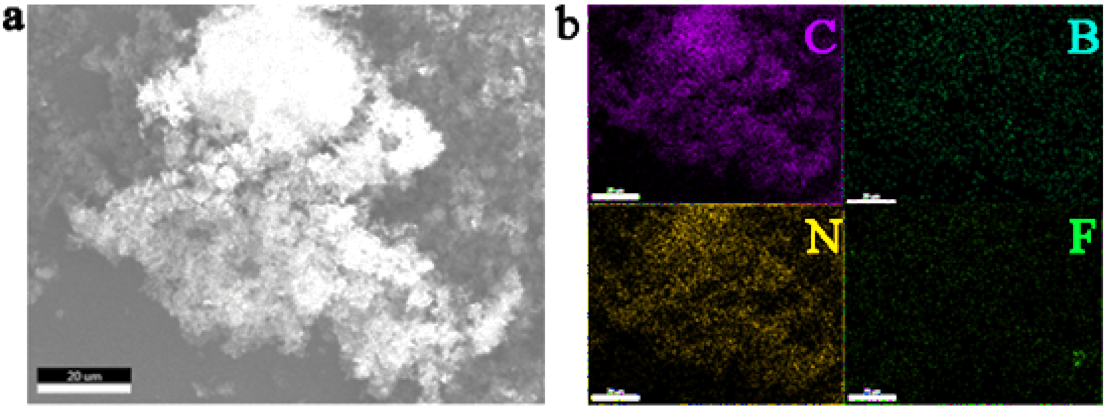

Figure S3. SEM (a) and EDS mapping image (b) of PAF-6@BCF.

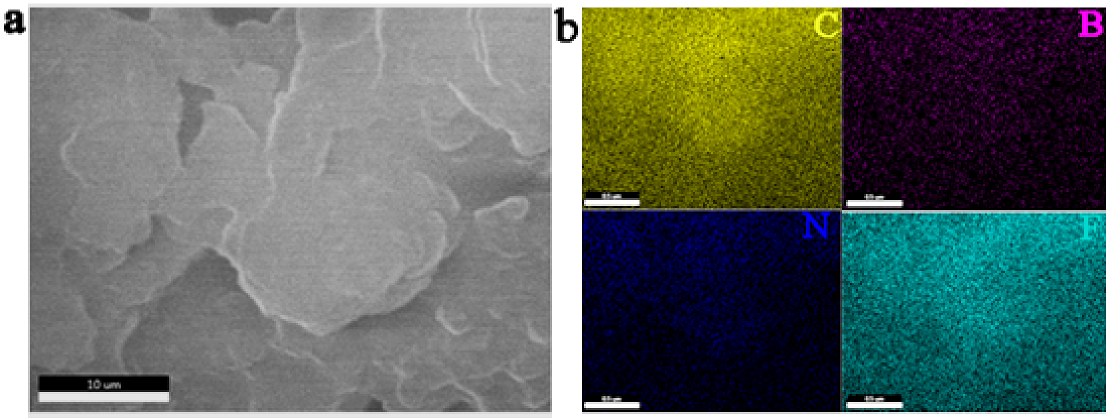

Figure S4. SEM (a) and EDS mapping image (b) of NanoPAF-6@BCF.

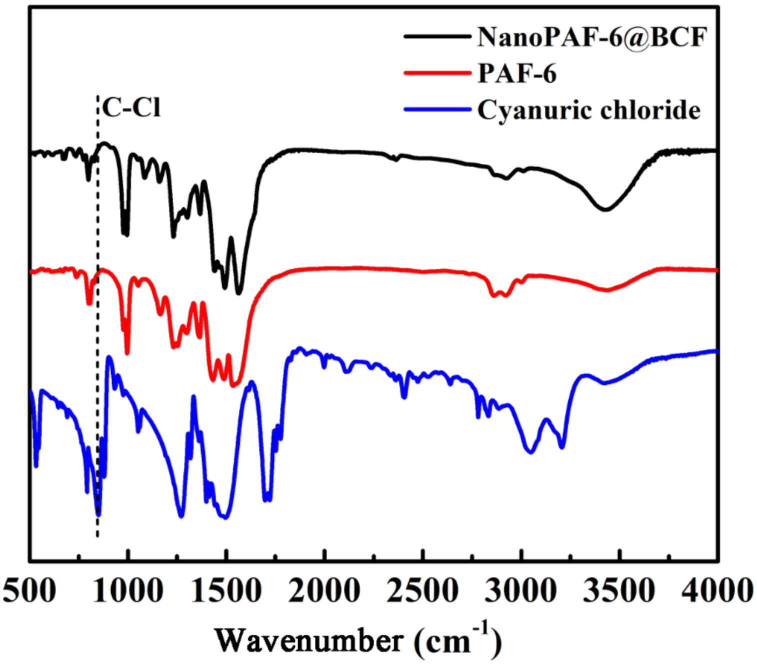

Figure S5. FTIR spectra of cyanuric chloride, PAF-6 and NanoPAF-6@BCF, respectively.

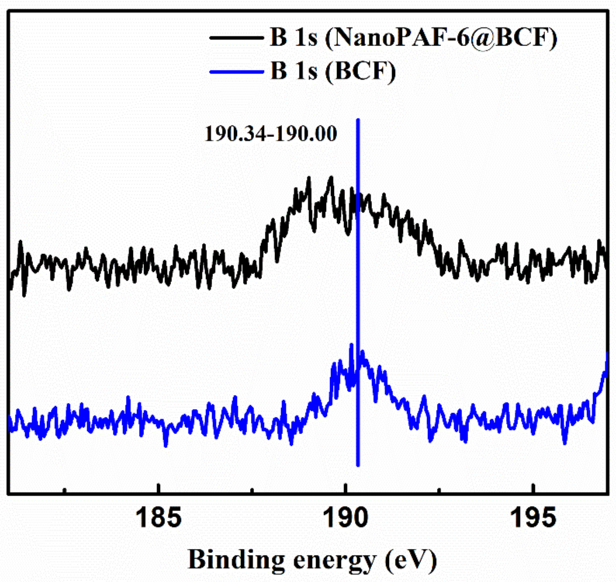

Figure S6. B 1s XPS spectra of NanoPAF-6@BCF and BCF.

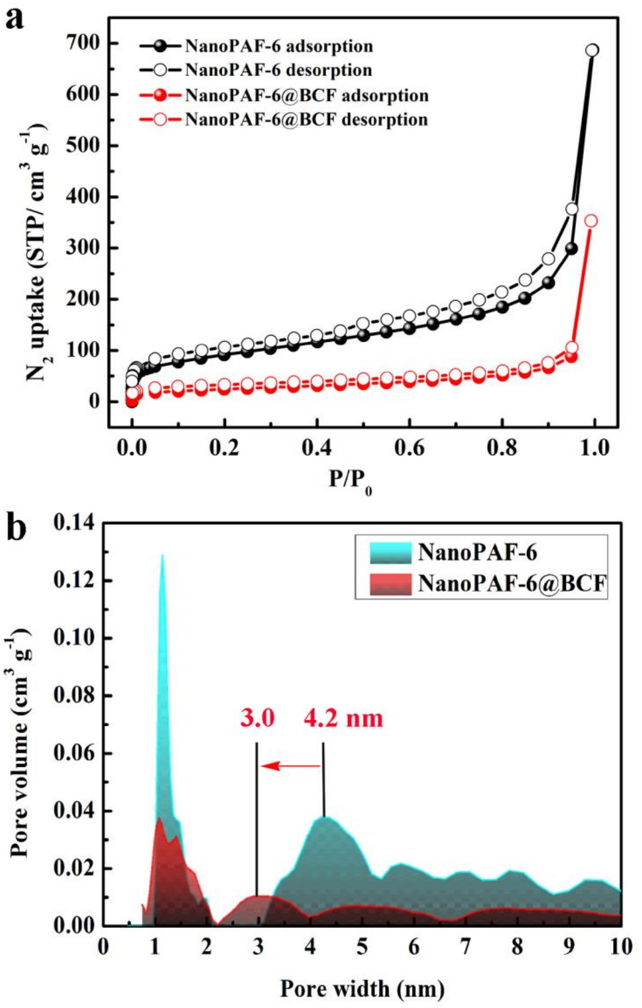

Figure S7. a) N_2 sorption isotherms and b) pore size distribution for NanoPAF-6 and NanoPAF-6@BCF.

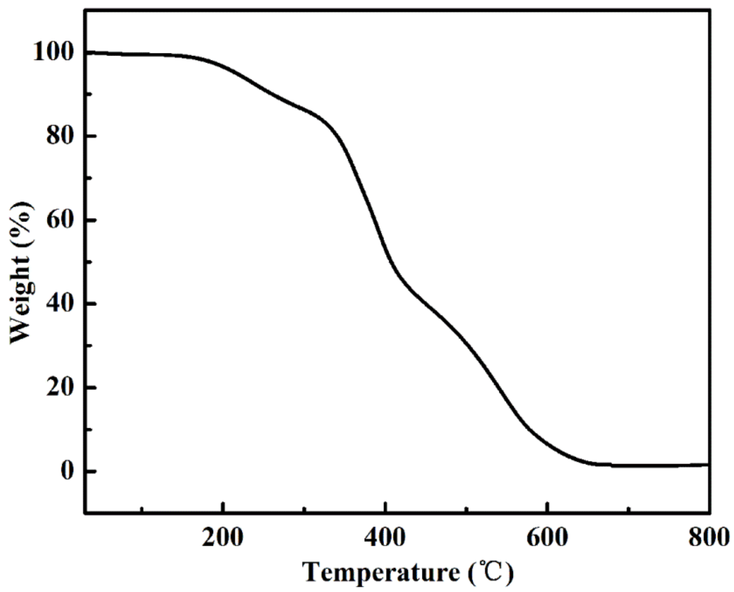

Figure S8. TGA curve of NanoPAF-6.

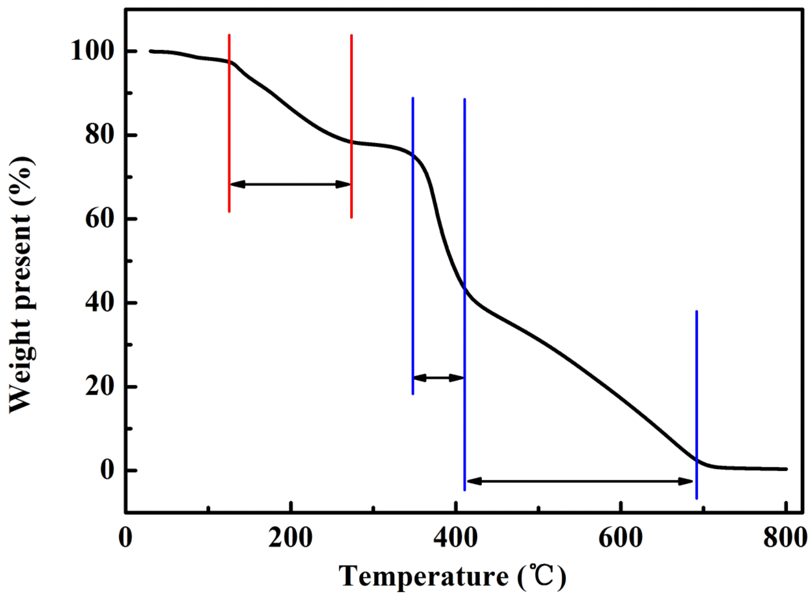

Figure S9. TGA curve of NanoPAF-6@BCF.

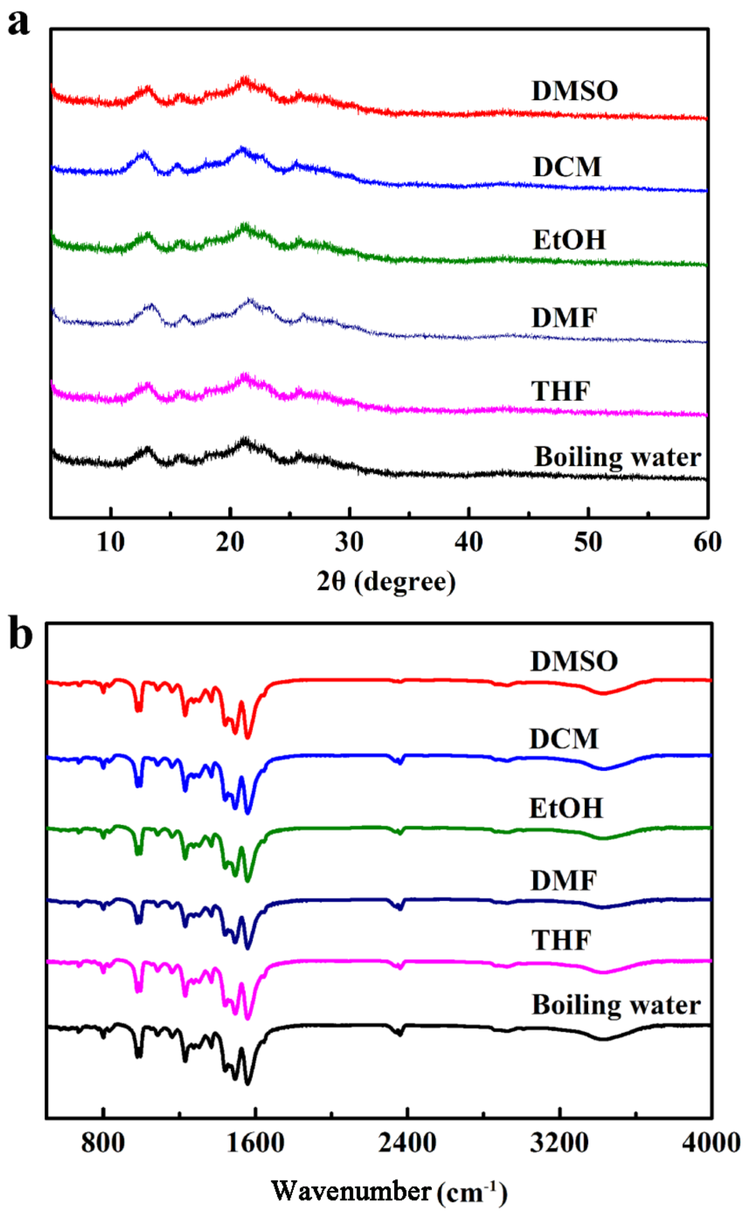

Figure S10. XRD patterns and FTIR spectra for NanoPAF-6@BCF after immersed into various solutions, respectively.

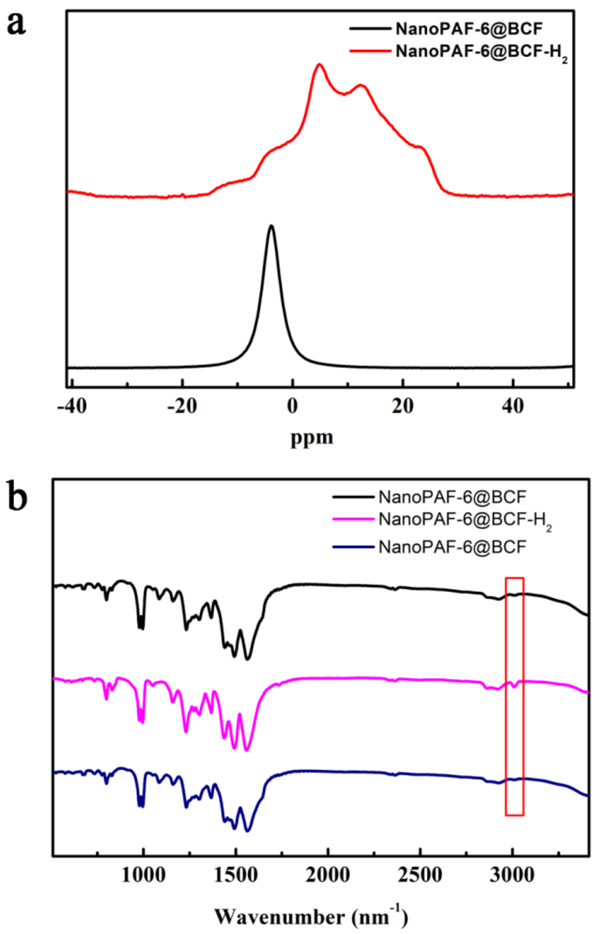

Figure S11. ^{11}B NMR (a) and FTIR (b) spectroscopy for NanoPAF-6@BCF and NanoPAF@BCF-H_2, respectively.

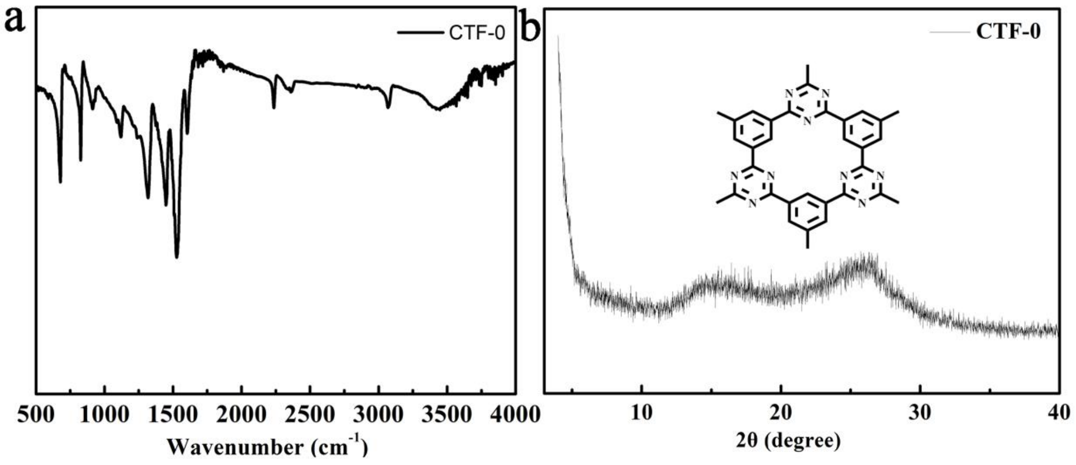

Figure S12. FTIR (a) and XRD (b) spectra of CTF-0.

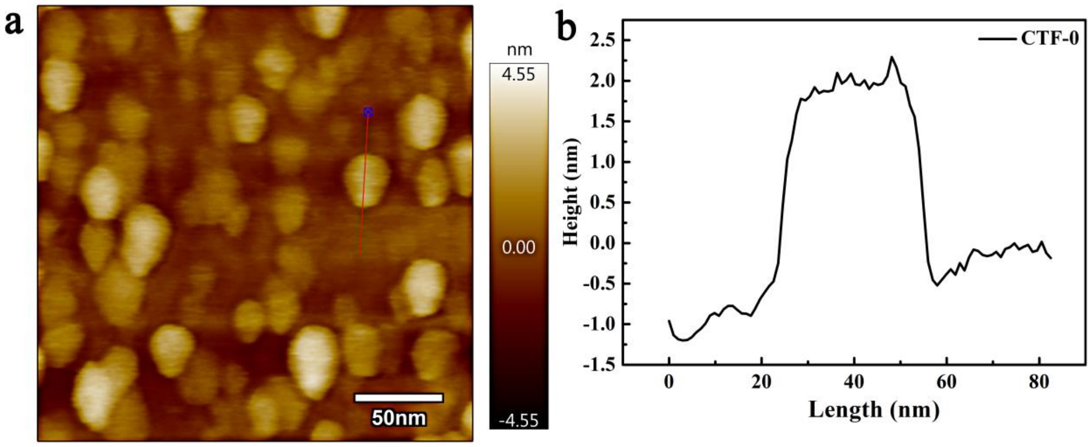

Figure S13. AFM image (a) and height profile (b) of CTF-0.

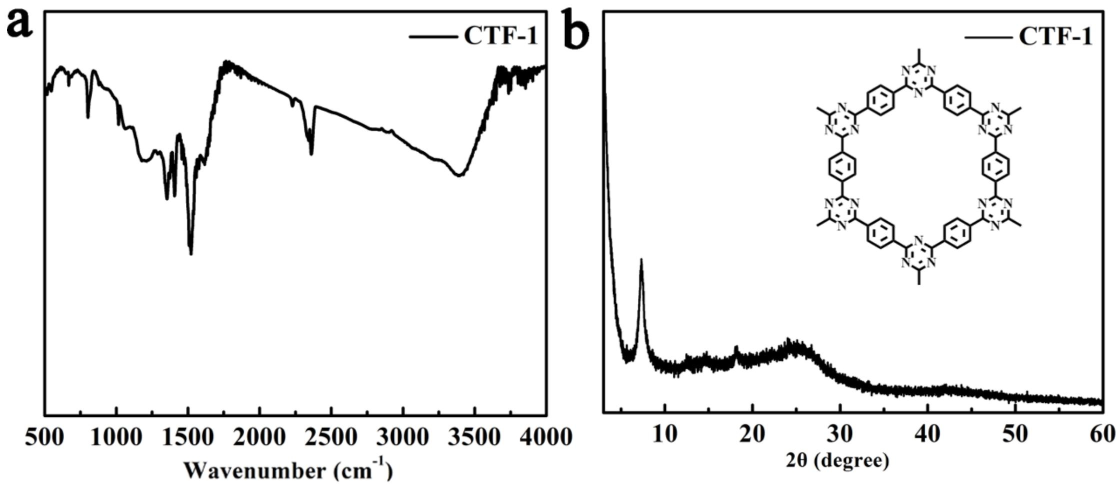

Figure S14. FTIR (a) and XRD (b) spectra of CTF-1.

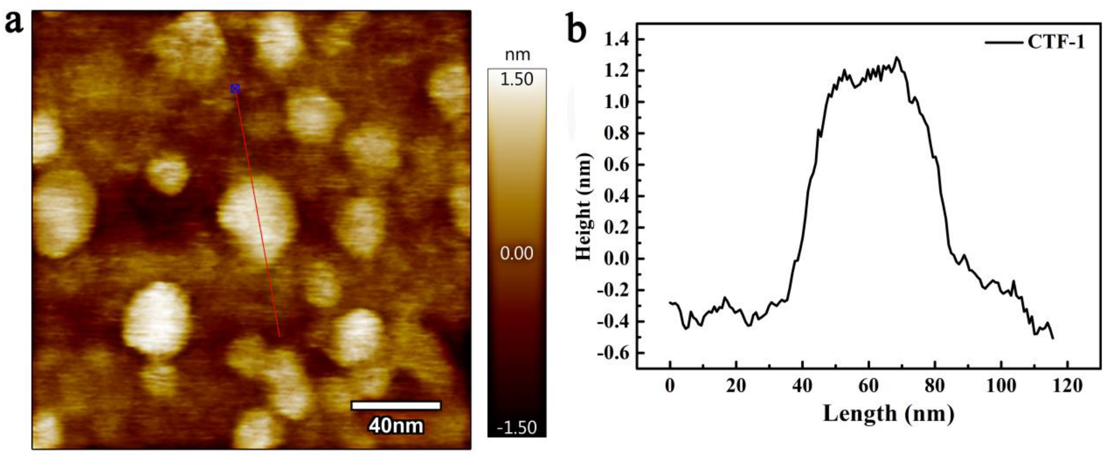

Figure S15. AFM image (a) and height profile (b) of CTF-1.

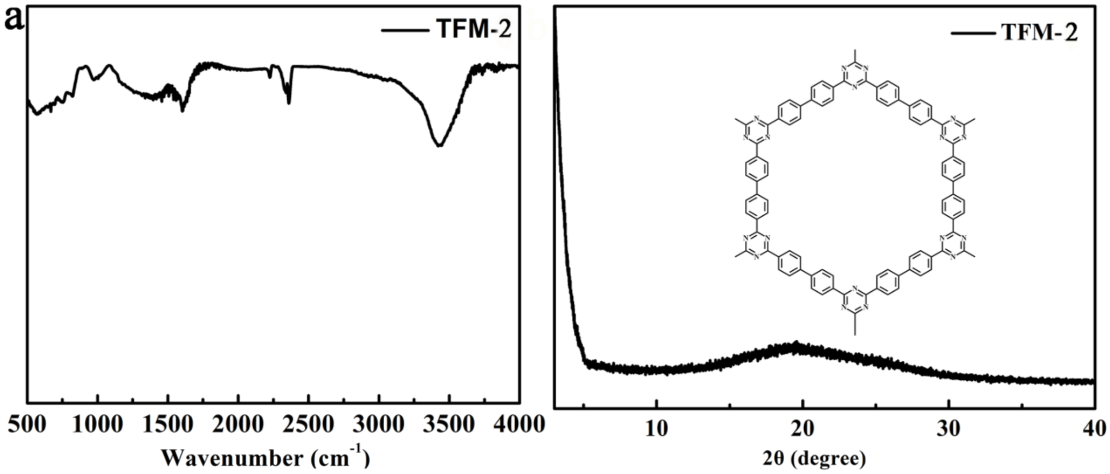

Figure S16. FTIR (a) and XRD (b) spectra of TFM-2.

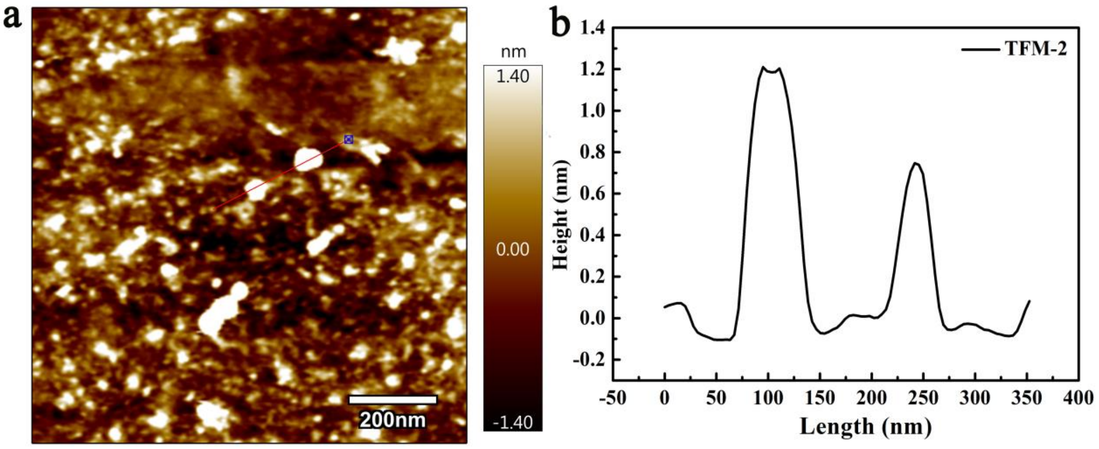

Figure S17. AFM image (a) and height profile (b) of TFM-2.

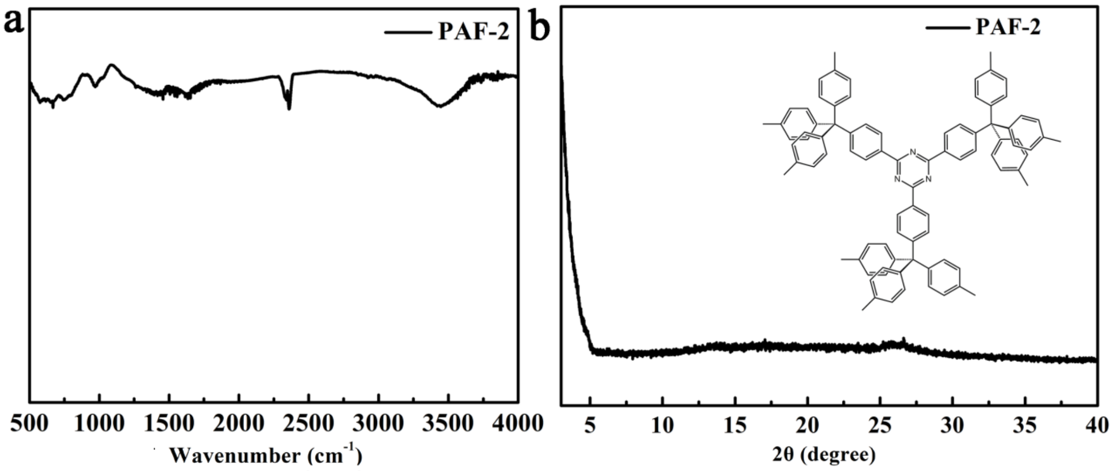

Figure S18. FTIR (a) and XRD (b) spectra of PAF-2.

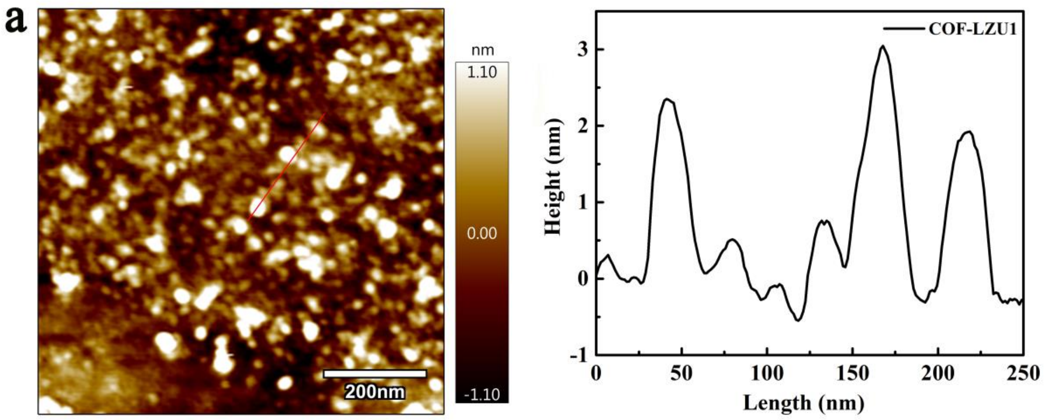

Figure S19. AFM image (a) and height profile (b) of COF-LZU1.

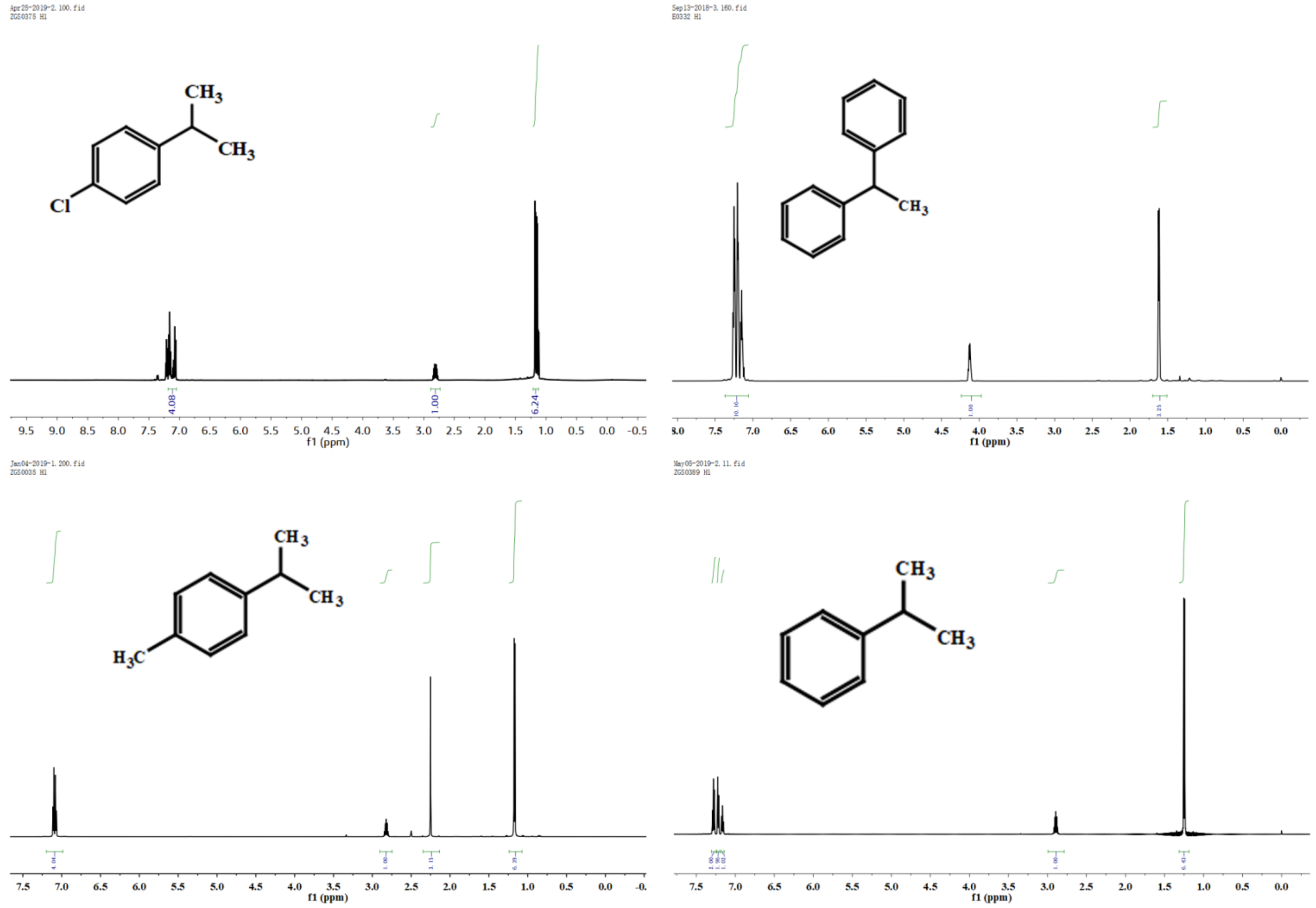

Figure S20. ^{1}H NMR spectra for various products catalyzed by NanoPAF-6@BCF.

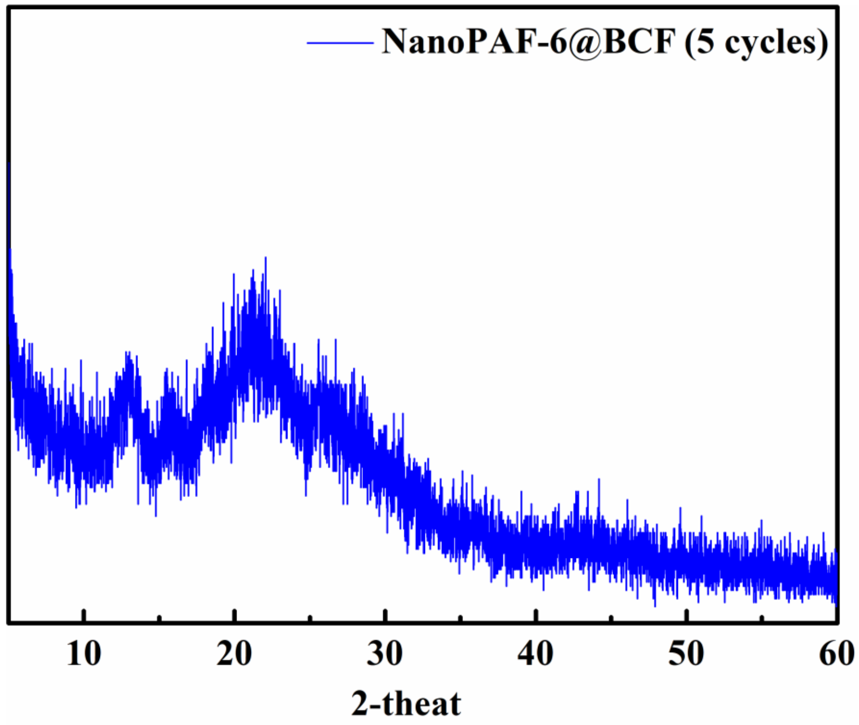

Figure S21. XRD image of NanoPAF-6@BCF after five cycles.

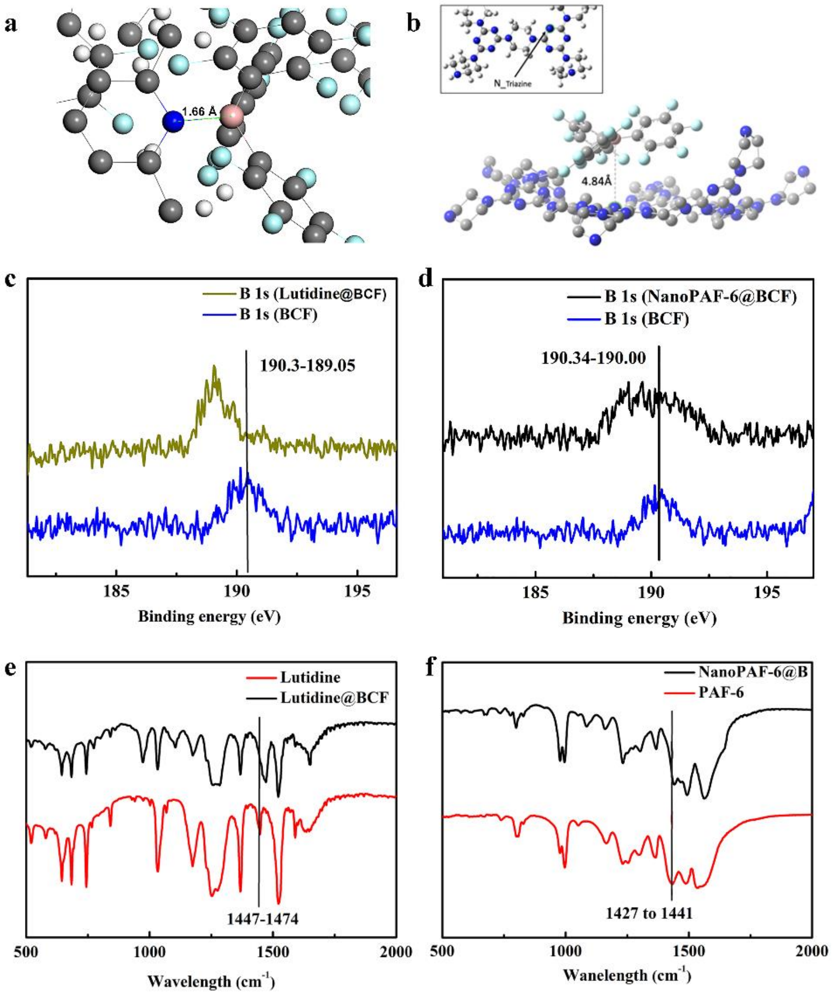

Figure S22. XPS and FTIR of BCF, lutidine@BCF and NanoPAF-6@BCF.

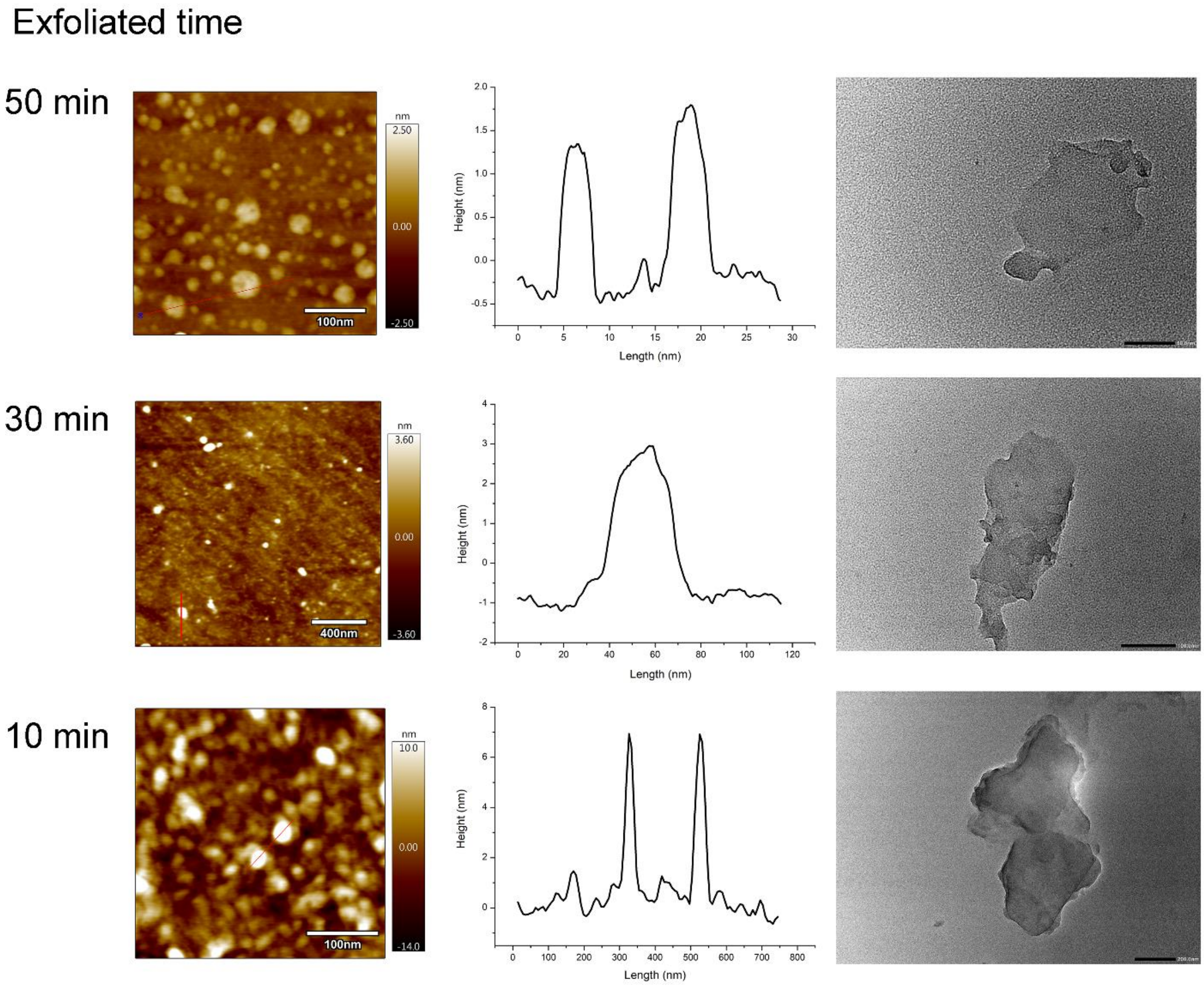

Figure S23. AFM image and TEM image of NanoPAF-6@BCF for different time.

Table S1. Catalytic activity of different structures.

Entry	Catalyst[a]	Pore size	N species	Yield (%)
1	BCF	--	--	0

2	1-Phenylpiperazine	-	$N_{Piperazine}$	0
3	1-Phenylpiperazine@BCF	-	$N_{Piperazine}$	0
4	2,4,6-Triphenyltriazine	-	$N_{Triazine}$	0
5	2,4,6-Triphenyltriazine@BCF	-	$N_{Triazine}$	0
6	PAF-6	1.2 nm	$N_{Piperazine}$ $N_{Triazine}$	0
7	NanoPAF-6			0
8	PAF-6@BCF			23
9	NanoPAF-6@BCF			91
10	CTF-0	0.5 nm	$N_{Triazine}$	0
11	CTF-0@BCF			0
12	NanoCTF-0@BCF			0
13	CTF-1	1.5 nm	$N_{Triazine}$	0
14	CTF-1@BCF			0
15	NanoCTF-1@BCF			0
16	TFM-2	2.0 nm	$N_{Triazine}$	0
17	TFM-2@BCF			0
18	NanoTFM-2@BCF			0
19	PAF-2	1.02 nm	$N_{Triazine}$	0
20	PAF-2@BCF			0
21	COF-LZU1	1.8 nm	$N_{Triazine}$ analogous	0
22	COF-LZU1@BCF			0
23	NnaoCOF-LZU1@BCF			0

[a] Each catalyst (~0.01 mmol of catalytic sites) was put into 5 mL dichloromethane (DCM) at air atmosphere and stirred for 10 min. Then 1,1-diphenylethene (0.2 mmol) was poured into the mixture, after purging with hydrogen flow for 5 min to remove the air the system was heated to 60 °C for 20 h under 10 bar H_2 atmosphere to measure the conversions.) b) Recycling performance of NanoPAF-6@BCF.

Table S2. Catalytic activity of both PAF and homogeneous N-B LP catalyst.

C_0 (mmol cm^{-3})	Q_e (mmol g^{-1})	C_v (mmol cm^{-3})	Yield (%)[a] PAF in C_0	C_v (mmol cm^{-3})	Yield (%)[b] N-B LP in C_v
0.001	0.01	0.05	21	0.05	19
0.01	0.06	0.28	61	0.28	58
0.02	0.09	0.43	71	0.43	72
0.03	0.12	0.57	82	0.57	79
0.04	0.13	0.62	91	0.62	89
0.06	0.13	0.62	91	--	--

[a]**Reaction conditions: NanoPAF-6@BCF (20 mg, 2.4 mmol cm^{-3}), 1, 1-diphenylethene (0.005-0.3 mmol are equivalent to 0.001-0.06 mmol cm^{-3}), DCM (5 mL), H_2 (10 bar),** 60 °C, 20 h. [b]**homogeneous N-B LP catalyst (12 mmol are equivalent to 2.4 mmol cm^{-3}), 1, 1-diphenylethene (0.25-3.10 mmol are equivalent to 0.05-0.62 mmol cm^{-3}), DCM (5 mL), H_2 (10 bar),** 60 °C, 20 h. **All conversions were measured by ^{1}H NMR integration and the product was purified by flash chromatography over silica gel to give the product.**

Table S3. Catalytic activity of different PAF thicknesses.

Exfoliated time (min)	Thickness (nm)	Q_e (mmol g^{-1})	B content	Reaction time (h^{-1})	Yield (%)[a]
non-exfoliated[b]	--	0.23	0.07 mg g^{-1}	20	23[b]
10[c]	7.06	0.19	1.05 mg g^{-1}	20	45[c]
30[d]	3.77	0.15	2.33 mg g^{-1}	20	73[d]
50[e]	1.95	0.13	4.64 mg g^{-1}	20	91[e]

[a]**General conditions: substrate (2 mmol), DCM (5 mL), H_2 (10 bar),** 60 °C, 20 h**. All conversions were measured by ^{1}H NMR integration and the product was purified by flash chromatography over silica gel to give the product.** [b]**PAF-6@BCF,** 20 mg; [c]**NanoPAF-6@BCF-12.66 nm,** 20 mg; [d]**NanoPAF-6@BCF-3.27 nm,** 20 mg; [e]**NanoPAF-6@BCF-1.95** nm, 20 mg.

Table S4. ICP data of NanoPAF-6@BCF of cycle experiment.

**Cycle times**	**B content**	**Yield**	**Cycle times**	**B content**	**Yield**
**1**	4.64 mg g^{-1}	90	**11**	4.55 mg g^{-1}	90

**2**	4.64 mg g^{-1}	91	**12**	4.54 mg g^{-1}	91
**3**	4.64 mg g^{-1}	89	**13**	4.53 mg g^{-1}	89
**4**	4.63 mg g^{-1}	90	**14**	4.51 mg g^{-1}	90
**5**	4.64 mg g^{-1}	92	**15**	4.47 mg g^{-1}	91
**6**	4.63 mg g^{-1}	90	**16**	4.43 mg g^{-1}	90
**7**	4.60 mg g^{-1}	91	**17**	4.41 mg g^{-1}	91
**8**	4.57 mg g^{-1}	93	**18**	4.38 mg g^{-1}	92
**9**	4.57 mg g^{-1}	90	**19**	4.34 mg g^{-1}	90
**10**	4.55 mg g^{-1}	92	**20**	4.32 mg g-1	91

References

[1] H. Zhao, Z. Jin, H. Su, X. Jing, F. Sun, G. Zhu, *Chem. Commun.* 2011, **47**, 6389.

[2] P. Katekomol, J. Roeser, M. Bojdys, J. Weber, A. Thomas, *Chem. Mater*. 2013, **25**, 1542.

[3] S. Y. Yu, J. Mahmood, H. J. Noh, J. M. Seo, S. M. Jung, S. H. Shin, Y. K. Im, I. Y. Jeon, J. B. Baek, *Angew. Chem. Int. Ed.* 2018, **57**, 8438.

[4] X. Zhu, C. Tian, S. M. Mahurin, S. H. Chai, C. Wang, S. Brown, G. M. Veith, H. Luo, H. Liu, S. Dai, *J. Am. Chem. Soc*. 2012, **134**, 10478.

[5] H. Ren, T. Ben, E. Wang, X. Jing, M. Xue, B. Liu, Y. Cui, S. Qiu, G. Zhu, *Chem. Commun.* 2010, **46**, 291.

[6] L. J. Hounjet, C. Bannwarth, C. N. Garon, C. B. Caputo, S. Grimme, D. W. Stephan, *Angew. Chem. Int. Ed.* 2013, **125**, 7640.

[7] L. Greb, P. OÇa-Burgos, B. Schirmer, S. Grimme, D. W. Stephan, J. Paradies, *Angew. Chem. Int. Ed.* 2012, **51**, 10164.
